# Homo- and Heterogeneous Benzyl Alcohol Catalytic Oxidation Promoted by Mononuclear Copper(II) Complexes: The Influence of the Ligand upon Product Conversion

**DOI:** 10.3390/molecules29112634

**Published:** 2024-06-03

**Authors:** Larissa Chimilouski, William H. Slominski, Ana I. Tillmann, Daniella Will, Aaron M. dos Santos, Giliandro Farias, Edmar Martendal, Karine P. Naidek, Fernando R. Xavier

**Affiliations:** 1 Departamento de Química, Centro de Ciências Tecnológicas (CCT), Universidade do Estado de Santa Catarina (UDESC), R. Paulo Malschitzky, 200 Zona Industrial Norte, Joinville 89219-710, SC, Brazilanatillmann12@gmail.com (A.I.T.); daniella.will@edu.udesc.br (D.W.);; 2 Departamento de Química, Centro de Ciências Física e Matemáticas, Universidade Federal de Santa Catarina (UFSC), R. Eng. Agronômico Andrei Cristian Ferreira, s/n, Trindade, Florianópolis 88040-900, SC, Brazil

**Keywords:** copper(II) complexes, benzyl alcohol oxidation, multivariate analysis, Fe_3_O_4_@SiO_2_ nanoparticles, catalysis

## Abstract

The catalytic properties of three copper complexes, [Cu(*en*)_2_](ClO_4_)_2_ (**1**), [Cu(*amp*)_2_](ClO_4_)_2_, (**2**) and [Cu(*bpy*)_2_](ClO_4_)_2_ (**3**) (where *en* = ethylenediamine, amp = 2-aminomethylpyridine and *bpy* = 2,2′-bipyridine), were explored upon the oxidation of benzyl alcohol (BnOH). Maximized conversions of the substrates to their respective products were obtained using a multivariate analysis approach, a powerful tool that allowed multiple variables to be optimized simultaneously, thus creating a more economical, fast and effective technique. Considering the studies in a fluid solution (homogeneous), all complexes strongly depended on the amount of the oxidizing agent (H_2_O_2_), followed by the catalyst load. In contrast, time seemed to be statistically less relevant for complexes **1** and **3** and not relevant for **2**. All complexes showed high selectivity in their optimized conditions, and only benzaldehyde (BA) was obtained as a viable product. Quantitatively, the catalytic activity observed was **3** > **2** > **1**, which is related to the π-acceptor character of the ligands employed in the study. Density functional theory (DFT) studies could corroborate this feature by correlating the geometric index for square pyramid Cu(II)-OOH species, which should be generated in the solution during the catalytic process. Complex **3** was successfully immobilized in silica-coated magnetic nanoparticles (Fe_3_O_4_@SiO_2_), and its oxidative activity was evaluated through heterogenous catalysis assays. Substrate conversion promoted by **3**-Fe_3_O_4_@SiO_2_ generated only BA as a viable product, and the supported catalyst’s recyclability was proven. Reduced catalytic conversions in the presence of the radical scavenger (2,2,6,6-tetrametil-piperidi-1-nil)oxil (TEMPO) indicate that radical and non-radical mechanisms are involved.

## 1. Introduction

Copper is found in many enzymes, biomolecules involved in electron transfer, the activation of oxygen and other small molecules such as methane and carbon monoxide, superoxide dismutation and even, in some invertebrates, oxygen transport [1]. Copper metalloenzymes are generally classified by their catalyzed reactions, substrate specificity and binding site [2]. These binding sites are distinguished based on standard biological copper centers. They are denoted as type I (T1 or blue copper), type II (T2 or normal copper), type III (T3 or binuclear copper) and trinuclear copper centers (TNC), which consist of a T2 and a T3 site [2,3]. Galactose oxidase (GOase) is a type II copper metalloenzyme with a molecular weight ranging from 65 kDa to 68 kDa. This enzyme catalyzes the oxidation of primary alcohols to the corresponding aldehydes with strict regioselectivity. Utilizing GOase is feasible in biosensors and chemical synthesis, in which various new mono-, oligo- and polysaccharide derivatives have been prepared [4]. Structurally, its active site contains a single copper center that adopts a distorted square pyramidal geometry. The copper center has five coordinating ligands: two tyrosines (Tyr272 and Tyr495), two histidines (His496 and His581) and a solvent molecule, usually water [5].

In parallel, mimicking the structural and functional properties of metalloenzymes remains a subject of great interest [6]. Among some works, the development of bioinspired metal complexes that can act as models for GOase has attracted much attention, and many of these compounds have been synthesized and characterized [7,8,9,10]. A typical organic substrate employed to test the GOase-like activity by model complexes is benzyl alcohol (BnOH). Copper complexes that can catalytically oxidize this substrate to benzaldehyde (BA) using different conditions, oxidizing agents and additives have been reported [11,12,13]. Industrially, BA is strategically relevant and finds applications in spices, pharmaceuticals, adhesives and dyes [14].

Although GOase and a significant portion of its bioinspired metal complexes usually have their catalytic activity evaluated in homogenous media, the development of heterogeneous catalysts employed in BnOH oxidation has been described. Inorganic platforms such as zeolites [15], noble metal nanoparticles [16,17,18], amorphous and mesoporous SiO_2_ [19], copper(II) dopped phyllosilicates [20], alkali-earth metal oxide [21], Al_2_O_3_ [22] and transition metal oxides [23,24,25] have been used in catalytic studies. Another particularly interesting material used to anchor catalysts is SiO_2_-coated magnetic Fe_3_O_4_ nanoparticles [26]. This approach is very convenient for rapidly separating/retrieving the catalyst from the mother solution by applying an external magnetic field when the catalytic process is over. Fe_3_O_4_ nanoparticles also present desirable features such as biodegradability, biocompatibility, practicality and low preparation cost [27]. Sarkheil et al. [28] described a copper(II) Schiff base complex immobilized on Fe_3_O_4_@SiO_2_, which was exceptionally efficient for norbornene and BnOH oxidation. Pyridinecarboxaldimine grafted onto magnetic Fe_3_O_4_@SiO_2_ NPs was prepared by Zhang and co-workers [29], then treated with CuBr_2_. In the presence of TEMPO and a base, selected primary alcohols were quickly aerobically oxidized to their respective aldehydes (yield > 90%).

Recently, we successfully described the study of catalytic oxidation of organic substrates through multivariate analysis employing the Box–Behnken statistical design [30,31,32]. This robust approach allowed us to maximize product conversion in catalyzed reactions by optimizing catalyst load, oxidant load and reaction time. This powerful tool also provided insights into how these variables were interconnected and influenced product conversion and selectivity. Here, we present the catalytic oxidation of benzyl alcohol (BnOH) mediated by mononuclear copper(II) complexes with *N*-rich bidentate ligands in homo- and heterogeneous media (silica-coated magnetic nanoparticles), aiming to understand the influence of these organic moieties upon product conversion.

## 2. Results and Discussion

### 2.1. Synthesis and Characterization

Complexes **1**, **2** and **3** were successfully prepared according to the methods already described, employing commercially available ligands [33,34,35]. For all complexes, the elemental analysis confirmed the [CuL_2_](ClO_4_)_2_ chemical composition, where L is ethylenediamine, 2-aminomethylpyridine and bipyridine for **1**, **2** and **3**, respectively. FTIR spectroscopy (Figure S1) showed that the ligands are present in all complexes, whereas an additional absorption around 1090 cm^−1^ could be observed and attributed to the perchlorate counterions in **1**, **2** and **3**. Characteristic vibrational modes such as NH stretching for **1** and **2** and C=C/C=N stretching found in **2** and **3** had their wavenumbers shifted to slightly lower energies. This fact is a good indicator that metal complexation has occurred. Finally, the out-of-plane angular deformation of the aromatic bonds (δ_CH_) in 2-aminomethylpyridine and 2,2′-bipyridine (ligands) is also present in the FTIR spectra of complexes **2** and **3**, respectively, confirming their presence in the copper complexes herein discussed. Molar conductivity corroborated the presence of ClO_4_^−^ ions in a 2:1 ratio, fully agreeing with the microanalysis herein reported. Electronic spectroscopy (Figure S2) was performed in acetonitrile solution, where typical copper(II) *d*–*d* transitions between 500 nm and 1100 nm, as well as charge transfer processes, were observed. A thorough UV-Vis discussion is described in the DFT section of this work. Finally, the electrochemical behavior of all complexes was evaluated using cyclic voltammetry (Figure S3). The Cu(II)/Cu(I) reduction processes observed at *E*_pc_ = −1.05 V, −0.64 V and −0.21 V vs. NHE for **1**, **2** and **3**, respectively, clearly reflected the increase in the ligand strength (ethylenediamine < 2-aminomethylpyridine < bipyridine), as well as its ability to stabilize the reduced species, thus increasing the process’ reversibility. Considering all data collected and presented in the Section 3, it was assumed that all complexes were successfully obtained.

The obtention, purity and particle size of all Fe_3_O_4_-based compounds were monitored through DLS, TEM and XRD techniques (Figures S4–S7). The **3**-Fe_3_O_4_@SiO_2_ nanocomposite was obtained by anchoring complex **3** in core–shell Fe_3_O_4_@SiO_2_ nanoparticles, with an anchorage rate of 5%. The **3**-Fe_3_O_4_@SiO_2_ particles have a size of 700 nm due to aggregation (DLS analysis, Figure S4, bottom), whereas the TEM images show individual particles of around 150 nm. The TEM images show a structure involving the light amorphous silica shell and the ligand that coated and wrapped the dark magnetic Fe_3_O_4_ (Figure S5). The XRD pattern of Fe_3_O_4_@SiO_2_ is illustrated in Figure S6. The characteristic diffraction peaks at 2θ = 30.23°, 35.55°, 43.23°, 53.54°, 57.20° and 62.64° were related to the (220), (311), (400), (422), (511) and (440) indices, respectively (JCPDS—Joint Committee on Powder Diffraction Standards—19-0629 standard data) [36]. These results correspond to the standard XRD pattern for Fe_3_O_4_ having the cubic spinel structure of the magnetite. Therefore, the crystal structure of the synthesized magnetic Fe_3_O_4_ nanoparticles remained unaffected during the synthesis of Fe_3_O_4_@SiO_2_. X-ray energy dispersive spectroscopy (EDS) was employed to determine the chemical composition of the **3**-Fe_3_O_4_@SiO_2_ nanocomposite (Figure S7). The resulting spectrum confirmed the presence of the elements C, O, Fe, Si and Cu (from the support grid) in the structure of **3**-Fe_3_O_4_@SiO_2_. All data suggest that **3**-Fe_3_O_4_@SiO_2_ was successfully obtained.

### 2.2. Homogeneous Catalytic Assays

**Figure 1:**
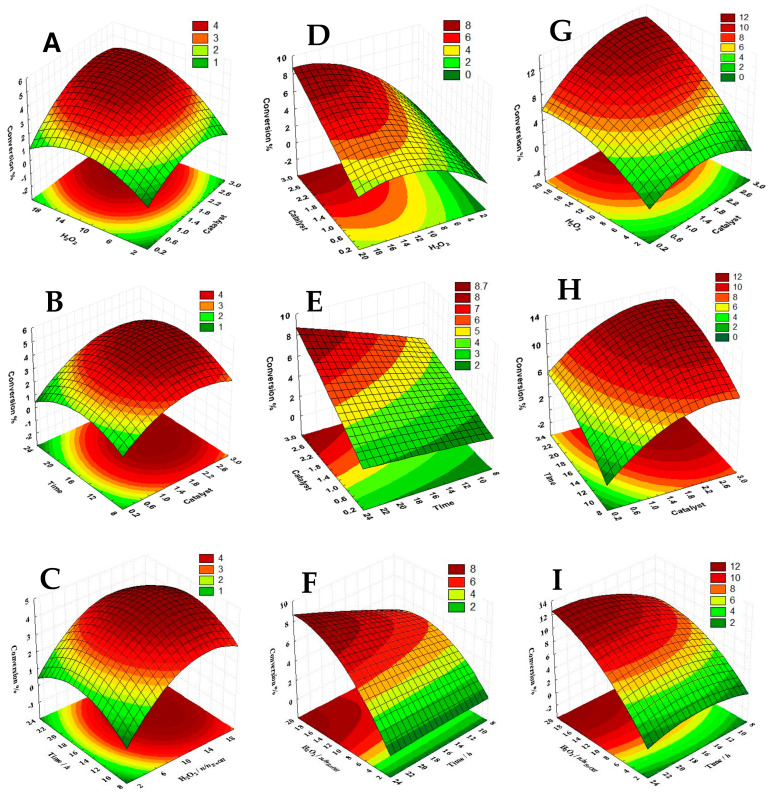
Response surfaces obtained by Box–Behnken’s experimental design for modeling BnOH to BA conversion (%) as a function of catalyst concentration, peroxide concentration and reaction time. Complex **1** (**A**–**C**, left), complex **2** (**D**–**F**, middle) and complex **3** (**G**–**I**, right). Each surface was generated with the missing variable at its optimal value. Optimal values for complex **1**: 14.5-fold excess H_2_O_2_ over substrate, 2.2 mol% as catalyst load and 16.3 h as the reaction time. Optimal values for complex **2**: 20-fold excess H_2_O_2_ over substrate, 3.0 mol% as catalyst load and 24 h as the reaction time. Optimal values for complex **3**: 20-fold excess H_2_O_2_ over substrate, 2.5 mol% as catalyst load and 24 h as the reaction time. Temperature was kept constant (20 °C).

The catalytic activity of complexes **1**, **2** and **3** was evaluated using the organic substrate benzyl alcohol (BnOH) in the presence of hydrogen peroxide as an oxidizing agent, using acetonitrile as a solvent. The products quantified were benzaldehyde (BA) and benzoic acid (BzA) obtained from the substrate conversion. Table 1 shows the intervals of the experimental conditions employed in the multivariate analysis and the conversion percentage obtained for each product (calculated from the calibration curve). The outcome data were inserted into Statistica 7 to obtain the response surfaces and the best reaction conditions (Figure 1).

**Figure 2:**
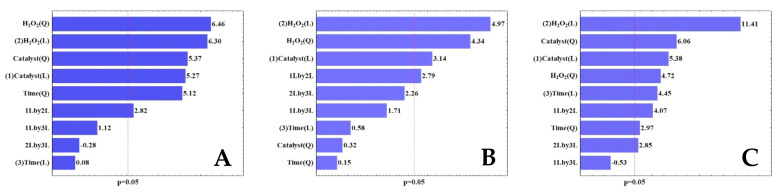
Response Pareto chart for the formation of BA promoted by **1** (**A**), **2** (**B**) and **3** (**C**). The values correspond to the effects of each coefficient of the Box–Behnken model. Effect values that exceed the dashed line are significantly greater than the experimental error at a 95% confidence level. In the graph along the y axis: L = linear; Q = quadratic; 1 = catalyst; 2 = H_2_O_2_; 3 = time; 1 L × 2 L = interaction between variables 1 and 2; 1 L × 3 L = interaction between variables 1 and 3; 2 L × 3 L = interaction between variables 2 and 3. The *x*-axis corresponds to the t-calculated value for each coefficient. When the t-calculated surpasses the critical t-value (*p* = 0.05), the coefficient is mathematically significant to the model.

For complex **1** (Figure 1A–C), a global optimal condition could be observed for the three factors evaluated, considering the conversion of the BnOH into BA. The catalyst load reached a plateau from 1.40 mol% up to 3.00 mol%, with an optimal value of 2.20 mol%. Excess hydrogen peroxide relative to the substrate had an optimized value of 14.5-fold, with the best reaction time of 16.3 h. All these variables were statistically significant when **1** was employed as a catalyst, as can be confirmed by the Pareto chart in Figure 2A, which infers that the linear and quadratic terms for the catalyst load, the quadratic and linear terms for the hydrogen peroxide concentration, as well as the reaction time and the interaction effect between the catalyst load and the hydrogen peroxide (1 L × 2 L) were significant (*p* > 0.05). Typically, when the interaction between two variables is significant, the optimal value for one is a function of the other, as can be seen in Figure 2A: as the catalyst load decreases, so does the optimal value for the H_2_O_2_ load. This suggests that the active species is an adduct between the catalyst and the H_2_O_2_. For the reaction time, only the quadratic term was shown to be significant, with a decrease in BnOH conversion into BA, suggesting that the active species can convert the formed BA into non-chromatographable components when the reaction time is far above the ideal level. In the optimized condition, the conversion of the BnOH into BA and BzA was 5.9% and 3.7%, respectively (Table 2, entry 1).

A local optimal condition was attained considering complex **2** (Figure 1D–F). This condition pointed to the maximum values for each factor evaluated (3.00 mol% catalyst load, 20 times more H_2_O_2_ than BnOH and a 24 h reaction time). The only variable that seems to be reaching a plateau is the H_2_O_2_ (see Figure 1D). The Pareto chart (Figure 2B) confirms what is seen on the response surfaces, as the only significant quadratic term is H_2_O_2_. In other words, no considerable curvature is observed on the response surfaces involving the reaction time and the catalyst load. On the other hand, linear terms for the H_2_O_2_ excess and the catalyst load were statistically significant; the higher their values, the higher the conversion rate. Considering variable interactions, the Pareto chart shows a slightly positive interaction between the catalyst load and the H_2_O_2_ concentration (1 L × 2 L, Figure 2B). This can be visualized in Figure 1D, in which the slope of conversion percentage in relation to the H_2_O_2_ concentration at 3.00 mol% of **2** is noticeably higher than that at 0.1 mol%. As for catalyst **1**, this represents marginal evidence that a plausible adduct can be formed between each metal complex, and H_2_O_2_ is the active species in this system. At the optimal local condition, the conversion rate of BnOH into BA and BzA was 7.75% and 5.24%, respectively (Table 2, entry 2).

When complex **3** is considered, response surfaces regarding its catalytic study are given in Figure 1G–I. Locally optimized values were determined for H_2_O_2_ and reaction time, and the highest levels evaluated were 20-fold excess of H_2_O_2_ over BnOH and 24 h. On the other hand, an optimal value for the catalyst load within the experimental domain (2.5 mol% of **3**) was detected. For this catalytic system, only the interaction between the catalyst load and reaction time was insignificant. All the quadratic terms were statistically relevant, corroborating the curvature seen in the response surfaces, especially for the H_2_O_2_ concentration and reaction time (Figure 1I). A significant interaction between the catalyst load and the oxidant excess is visualized in Figure 1G, which shows that, at a low hydrogen peroxide concentration, the conversion percentage remains almost unchanged and at a low level, regardless of the catalyst load. The same behavior is observed in Figure 1I for the interaction between the H_2_O_2_ concentration and the reaction time. At the optimized condition, the conversion percentage of BnOH into BA and BzA was 11.2% and 3.28%, respectively (Table 2, entry 3).

Taking the optimized conditions into account (Table 2, entries 1–3), fair selectivity was observed in benzaldehyde (BA) production, where complex **3** was the most active (higher conversion) and selective among them (78%). Its higher catalytic activity was also corroborated by its turnover number (TON), which was 65% and 73% higher than that of complexes **1** and **2**, respectively. Interestingly, when the catalytic activity was observed from the turnover frequency point of view (TOF, h^−1^), complex **3** still presented better performance than complexes **1** and **2** (10% and 72% higher, respectively), whereas the TOF of **1** was 57% higher than **2,** despite its higher conversion values (5.94 ± 0.27 and 7.75 ± 0.28 for **1** and **2**, respectively).

Considering the work reported by Vailati and coworkers [30], where a copper(II) complex containing a polypyridyl ligand had its catalytic ability to convert BnOH into BA tested in similar conditions, the same strong dependence on H_2_O_2_ excess and the catalyst load, as well as the less pronounced effect of time, was observed. On the other hand, for the dinuclear copper(II) complex with a polypyridyl bioinspired ligand and a pyrazole-bringing exogeneous ligand described by Chimilouski and collaborators [32], all three significant variables (catalyst load, H_2_O_2_ excess and time) seemed to be relevant to maximize BA production. 

Table 1 shows that, in several entries, no BzA formation is present. Thus, response surfaces concerning this reaction product could not be plotted due to the lack of sufficient experimental data. However, BzA was formed primarily when the reaction time was at the highest level evaluated, except for some entries such as 2 (complex **1**), 4 (complexes **1** and **3**), 12 (all complexes) and entries 13 to 15 for complexes **1** and **2**. Fortunately, in the optimized conditions for all complexes, the conversion of the BnOH into its respective oxidation products, both BA and BzA, was detected and thoroughly quantified.

Table 2 shows, in brackets, the values predicted from the mathematical modeling for the conversion of the BnOH into BA. An average agreement of 102% between experimental and predicted values indicates the excellent accuracy of the prediction model. Furthermore, the optimal conditions and maximum BA conversion obtained by complex **3** (Table 2, entry 3) are similar to those reported by Vailati and coworkers (2.75 mol% of catalyst load, 18-fold H_2_O_2_ over substrate and 20 h) [30].

A set of assays were performed to rationalize the catalytic system (Table 2, entries 4 to 14). At the optimized condition for each complex, a triplicate experiment was carried out using Cu(ClO_4_)_2_·6H_2_O as a control (entries 4 to 6). Complexes **1**, **2** and **3** were able to convert BnOH into BA by a factor of 1.41, 1.04 and 1.46 when compared to the use of the “bare” copper salt as a catalyst. This is an important indication that the ligands could stabilize their respective active species formed during the reaction. Additionally, using the copper salt did not yield BzA under the evaluated conditions.

Only low amounts of BA were detected when no catalyst was employed during the reaction tests, considering the optimized catalytic conditions (Table 2, entries 7 and 8). This indicates that the copper complexes are crucial to BnOH’s conversion into products.

Interesting findings could be observed when BA was used as a substrate instead of BnOH (Table 2, entries 9 to 11). The conversions of BA into BzA were 93.6%, 71.6% and 56.2% for complexes **1**, **2** and **3**, respectively. This trend suggests that the catalytic activity regarding the conversion of BA into BzA decreases from complex **1** to **3**. Conversely, when BnOH was the substrate, catalytic activity was in the opposite direction. The difference in this behavior will be addressed in the DFT section of this work. These higher yields obtained when converting BA into BzA imply that BnOH is much less reactive than BA (as expected), and the reaction to afford the BzA species is sequential: from BnOH to BA, and then from BA to BzA. Control experiments where no catalyst was employed during the oxidation of BA to afford BzA were carried out, and no product formation was observed (entries 12 and 13).

Finally, we performed the reaction by adding TEMPO (4 eq.), a known general capturer of free radicals, using BnOH as the substrate for each complex under their optimized reaction conditions (Table 2, entries 14 to 16). The conversions decreased by an average of 57% compared to those reactions where no TEMPO was used. These findings suggest that radical species were formed during the reactions catalyzed by complexes **1**, **2** and **3**. Considering that substantial conversions were observed even in the presence of the radical scavenger TEMPO, more than one mechanistic pathway is involved in the catalytic oxidation of BnOH promoted by the complexes herein studied. Similar TEMPO tests were carried out when BA was used as the substrate (Table 2, entries 17 to 19). Both complexes **1** and **2** had their conversions from BA to BzA diminished, indicating that radical species were involved during the reactions. Complex **3** presented a slight increase in conversion rate (around 10%); thus, no radical mechanistic pathways were involved in this case.

### 2.3. Heterogeneous Catalytic Assays

Once complex **3** presented the best catalytic performance in a fluid solution (homogeneous media), it was successfully anchored in silica-covered magnetic nanoparticles to produce the composite **3**-Fe_3_O_4_@SiO_2_, and then was tested as a heterogeneous catalyst. To assess its activity in the conversion of BnOH into BA, the optimized catalytic parameters found for the homogeneous study (Table 2, entry 3) were adapted, reducing the catalyst load to 1.0 mol% due to the low immobilization of **3** into Fe_3_O_4_@SiO_2_, which would lead to a large amount of **3**-Fe_3_O_4_@SiO_2_ per catalytic run to compensate.

Considering these parameters, the BA production was 2.89%, significantly lower than the homogenous assays (11.2%). To improve the product formation, a new parameter, the reaction volume, was considered. Similar experiments employing a 1.0 mol% catalyst load, 20 times as much H_2_O_2_ as BnOH and a reaction time of 24 h were carried out with three different reaction volumes (Table 3, entries 1 to 3), and a linear trend could be observed with the conversion percentage as a function of the reciprocal of the reaction volume (mL^−1^) (Figure S8). As expected, the smaller the reaction volume, the more conversions were observed. In this sense, all heterogeneous catalytic studies were performed with 3.0 mL of the solution. The conversion of BnOH into BA promoted by **3**-Fe_3_O_4_@SiO_2_ with these new parameters was 5.46% (an 89% increase), close to the homogeneous reaction with the same conditions (7.34%, Table 3, entry 4).

For all heterogeneous studies, the composite **3**-Fe_3_O_4_@SiO_2_ presented an outstanding selectivity (100%) towards benzaldehyde (BA) where no benzoic acid (BzA) was detected during the catalytic studies. When the turnover number (TON) for **3**-Fe_3_O_4_@SiO_2_ (Table 3, entry 1) is compared to the similar rection in homogeneous conditions (Table 3, entry 4), the supported catalyst presented only a 25% decrease in catalytic activity. When the reaction time was 24 h for both catalysts, the same reduction in the catalytic performance in terms of turnover frequency (TOF, h^−1^) was observed for homo- and heterogeneous media.

As a negative control, the silica-covered magnetic nanoparticles (Fe_3_O_4_@SiO_2_) without complex **3** immobilized on their structures had their catalytic activity tested under the same catalytic parameters, and only a residual conversion was observed (Table 3, entry 5), thus proving that complex **3** is crucial for the catalysis. Finally, TEMPO (4 eq.) was added using BnOH as substrate (Table 3, entry 6). Surprisingly, the conversion of BnOH into BA catalyzed by **3**-Fe_3_O_4_@SiO_2_ was not affected (5.48% vs. 5.46% in the presence of the radical scavenger). This fact suggests that radical species were not involved during the reaction.

**Figure 3:**
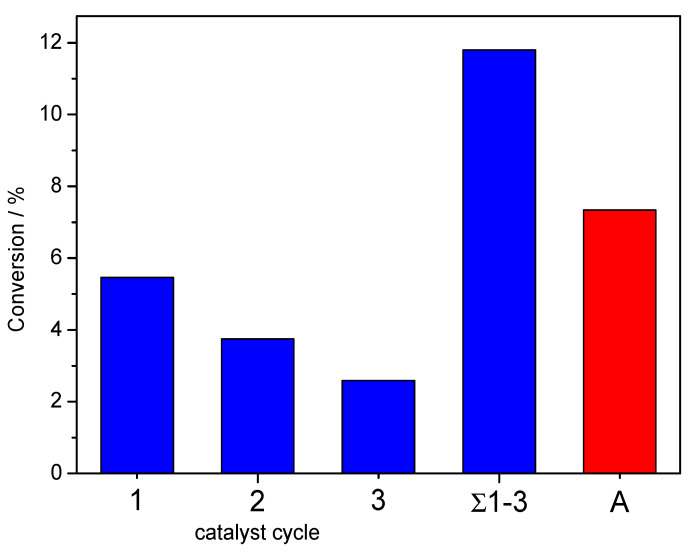
Blue: First use (1) and two reuses (2 and 3) of **3**-Fe_3_O_4_@SiO_2_ during the catalytic reactions to convert BnOH into BA, and the total conversion obtained from them (Σ1–3). Red: BA production from an equivalent reaction in the homogeneous medium with complex **3** (A). Conditions: 1.0 mol% of catalyst, 20-fold H_2_O_2_ over BnOH, 24 h, 20 °C, final volume 3.0 mL.

Once the **3**-Fe_3_O_4_@SiO_2_ catalyst displayed an assertive magnetic behavior, separating the catalyst from the rection solution using an external strong neodymium magnet was straightforward. In this sense, its recyclability was evaluated, and two more reuses were performed (Figure 3). During this process, a clear decrease in BA production was observed, which could be related to complex **3** degradation or solvent leaching. However, when the overall conversion was considered (Σ1–3), the production of BA could reach 11.8%, which is 60% higher than the homogeneous studies with the optimized conditions (7.34%, Figure 3, bar A (in red)).

### 2.4. Theoretical Insights

To gain a better understanding of the catalytic behavior of complexes **1**, **2** and **3** in converting BnOH into BA and BA into BzA, we conducted DFT calculations within the D3-B3LYP/def2-TVZP(-f) level of theory. At first, we obtained the ground state geometry of complexes **1**, **2** and **3** and compared the bond lengths, angles and IR frequencies with those obtained experimentally to validate the use of the chosen level of theory. As Tables S1 and S2 show, the theoretical values are in good agreement with the experimental ones, supporting the use of the D3-B3LYP/def2-TVZP(-f) level of theory in the subsequent investigations. Also, we used TD-DFT calculations to simulate the theoretical absorption spectra (Figure S9) and obtain information about the 30 lowest energies and allowed transitions. Comparing the theoretical and experimental spectra, one can attribute the first absorption band observed experimentally to four d–d* transitions in the metal center, as detailed in Tables S3–S5. The second and intense absorption band experimentally observed between 250 nm and 350 nm for **1**, **2** and **3** is assigned to charge transfer transitions between the ligands and the Cu(II) center. For complex **1**, this transition is only due to LMCT transitions from the coordinated amines to the half-filled *d*x^2^-y^2^ of metal Cu(II). For complex **2**, further MLCT transitions, mainly from *d*x^2^-y^2^ of metal Cu(II) to π* from pyridine and LMCT transitions from π orbitals of pyridine to Cu(II) *d*x^2^-y^2^ orbital arise with energies close to those of n(NH) → *d*x^2^-y^2^ LMCT and add up to a more intense absorption band, as also observed experimentally. For complex **3**, mainly π(pyridine) → *d*x^2^-y^2^ LMCT and π(pyridine) → π*(pyridine) are observed in this region and, due to the larger possibilities of transitions with a moderate oscillator strength with close energy, an intense band arises in this region, also in agreement with the UV-Vis experiment.

**Figure 4:**
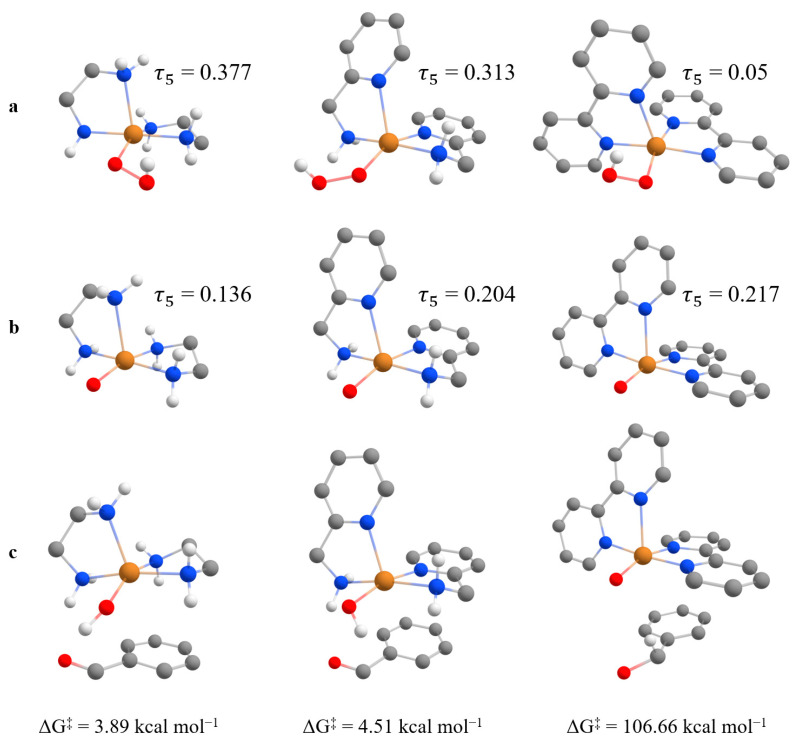
Optimized ground state geometry for Cu(II)-OOH species (**a**), [Cu(II)-O•]^+^ species (**b**) and the TS state for the H• transfer between [Cu(II)-O•]^+^ species and BA (**c**) obtained within the D3-B3LYP/def2-TVZP(-f) level of theory.

To discuss the catalytic behavior, we considered previous studies concluding that the reactivity of mononuclear Cu complexes with H_2_O_2_ is mainly governed by the coordination geometry around the metal center. Mononuclear complexes that generated a Cu(II)-hydroperoxo intermediate with a square pyramidal geometry exhibited substrate oxidation, while those with a trigonal bipyramidal geometry displayed no reactivity [37]. Therefore, the ground state geometry for Cu(II)-OOH species of complexes **1**, **2** and **3** was obtained (Figure 4a) to evaluate the geometry around the metal center through the geometry index $\tau_{5}$ [38], and we tried to correlate it with the catalytic conversions. All possibilities of isomers for **2** and conformers for the Cu-O bond rotation in **1**, **2** and **3** were evaluated and only the stable ones are considered in the discussions. The obtained geometry index follows the order **1** ($\tau_{5}$ = 0.377) > **2** ($\tau_{5}$ = 0.313) > **3** ($\tau_{5}$ = 0.05). Considering that a perfect square pyramidal geometry ($\tau_{5}$ = 0.00) would show higher catalytic activity, the order agrees with the yield of the BnOH to BA conversion, with complex **3** having a higher conversion. Singh and co-workers [37] studied the oxidation mechanism for similar compounds. They observed that the reaction-determinant step (RTP) when Cu(II)-OOH species are involved is the breaking of the Cu-O bond. In this case, there is no radical species before the RTP. This agrees with the similar yield decrease observed when adding TEMPO to these reactions, as it kills radicals later than the RTP, similarly affecting the reactions for all complexes.

For the conversion from BA to BzA, an inversed yield in the order **1** > **2** > **3** was experimentally observed. This trend does not agree with the geometry index parameter for Cu(II)-OOH and can be related to the substrate oxidation by [Cu(II)-O•]^+^ species. To these [Cu(II)-O•]^+^ species derived from complexes **1**, **2** and **3**, the ground state geometry and the geometry index $\tau_{5}$ were also obtained (Figure 4b). In this case, a reverse order to the geometry index **3** ($\tau_{5}$ = 0.217) > **2** ($\tau_{5}$ = 0.204) > **1** ($\tau_{5}$ = 0.136) was also obtained, with **1** showing a geometry closer to a square pyramid in agreement with the observed BA to BzA yield.

Adding TEMPO significantly decreases the conversion for **1** and **2** due to the presence of [Cu(II)-O•]^+^. At the same time, for **3**, a slight increase is observed. To further evaluate this, we obtained the free energy of activation (ΔG^‡^) for the radical proton transfer reaction between [Cu(II)-O•]^+^ and BA, considering this to be the first step for BA oxidation. Figure 4c shows the transition state geometry and the ΔG^‡^ values. For **1** and **2**, the ΔG^‡^ agrees with **1**, showing a higher conversion with a lower activation energy. However, the activation energy for **3** is relatively high, which may indicate that the oxidation mechanism goes through Cu(II)-OOH species instead of [Cu(II)-O•]^+^. Thus, if, for **3**, the oxidation goes through a mechanism where the RTP does not contain a radical species, this agrees with the reaction being unaffected by adding TEMPO only for this complex.

Based on the results obtained in this work for the conversion rate of BnOH into BA and BzA, a direct comparison with other studies in the literature that use the same substrate and oxidizing agent is not possible due to the lack of methodological information on how the conversions were determined and calculated. To ensure accurate determination, authentic standards of the reaction products must be used, at least the main ones, rather than simply stating that the conversion was determined via gas chromatography. The quantification of the products in this work was carried out properly using BnOH, BA and BzA standards. During the process, we suggested the presence of non-chromatographable substances, which may be attributed to the formation of highly polar compounds or even mineralization. Not all the initial amount of BnOH, in moles, was converted into BA and BzA, and the chromatogram did not reveal any other compounds. The normalization method, which considers the sum of the peak area of the products related to a 100% conversion, cannot be used to calculate the rate of conversion of BnOH into BA and BzA via the reaction with hydrogen peroxide catalyzed by metal complexes, as is the case of this work and other studies in the literature. Furthermore, if we had not carried out the quantification carefully, using the normalization method instead, we would have found unrealistic conversion percentages of over 90% for BA and over 9% for BzA. It is important to note that these percentages are not accurate and should not be used for any further analysis.

## 3. Experimental Procedures

### 3.1. Materials and Instruments

Copper perchlorate hexahydrate, 2-aminomethylpyridine, 2,2′-bipyridne and tetraethyl orthosilicate (TEOS) were purchased from Sigma-Aldrich (St. Louis, MO, USA). Benzyl alcohol, benzaldehyde, benzoic acid, hydrogen peroxide, ethylenediamine, methanol, acetonitrile, iron(II) sulfate, potassium nitrate and potassium hydroxide were purchased from Dinânica (Indaiatuba, SP, Brazil). The chemicals 2-aminomethylpyridine, benzaldehyde, ethylenediamine and acetonitrile were distilled under reduced pressure before use. All others were used as received.

Elemental analysis determinations (C, H and N) were performed on a Perkin-Elmer 2400 CHNS/O Series II analyzer (Waltham, MA, USA). Infrared spectra were collected on a Bruker INVENIO-S spectrometer (Ettlingen, KA, Germany) using the ATR module in the 4000 cm^−1^ to 400 cm^−1^ range with a 4 cm^−1^ resolution. The data were plotted as transmittance (%) as a function of wavenumber (cm^−1^). The molar conductivity was evaluated in an MS-Tecnopon mCA150 conductivity meter (Piracicaba, SP, Brazil), previously calibrated with an aqueous 1.00 × 10^−2^ mol L^−1^ KCl solution; Λ_M_ = 1408 μS cm^−1^ [39] at 25 °C, using 1.00 × 10^−3^ mol L^−1^ spectroscopic grade acetonitrile solutions. The electrochemical behavior of all complexes was investigated with a GAMRY 1010E potentiostat/galvanostat (Warminster, PA, USA). Cyclic voltammograms were obtained at room temperature in acetonitrile solutions containing 0.1 mol L^−1^ of *n*-Bu_4_NPF_6_, a supporting electrolyte under an argon atmosphere. The electrochemical cell comprised three electrodes: platinum (working), platinum wire (auxiliary) and Ag/Ag^+^ (reference). The internal standard was a ferrocene/ferrocenium redox couple Fc/Fc^+^ (*E*^o^ = 400 mV vs. NHE) [40]. All potentials herein described were referenced towards the normal hydrogen electrode (NHE). UV-visible spectra were obtained at room temperature using a Shimadzu UV3600 Plus spectrophotometer (Kyoto, Japan) operating in the 180 nm to 1300 nm range with quartz cells. Values of molar absorptivity, ε, were given in L mol^−1^ cm^−1^. The spectra were collected in acetonitrile and the solid state with a 60 mm diffuse reflectance integrating sphere (ISR-603).

The Fe_3_O_4_-based materials were analyzed in a JEM 2100 (JEOL, Tokyo, Japan) transmission electronic microscope (TEM) and through X-ray energy dispersive spectroscopy (EDS), with samples previously dispersed in acetone and sonicated for 2 min. The X-ray powder diffraction analysis was performed using a D2 Phaser diffractometer, using Bragg–Brentano geometry (Bruker, Karlsruhe, Germany), equipped with a LynxEye detector and operated through a Cu Kα source (1.5418 Å) at 30 kV and 10 mA. The data were collected in the 2θ interval from 20° to 80° with increments of 0.04° and a scan time of 1.5 s per step. The size distribution was also obtained through dynamic light scattering (DLS) using a Litesizer 500 (Anton Paar, Graz, Austria)—from aqueous dispersed samples sonicated for 5 min.

### 3.2. DFT Methodology

The geometry optimization of derivatives from complexes **1**, **2** and **3**, Cu(II)-OOH and [Cu(II)-O•]^+^, was performed at the density functional theory (DFT) level using the B3LYP [41,42] and def2-TZVP(-f) basis set for all atoms [43,44,45]. Dispersion effects (if present) were included using Grimme’s D3 correction with Becke–Johnson (BJ) damping [46,47]. The evaluation of the four-center integrals was accelerated with the RIJCOSX algorithm, using the resolution-of-the-identity approximation for the Coulomb part (RIJ) and the chain of spheres approach for the Fock exchange (COSX) [48,49]. RIJ requires the specification of an auxiliary basis set for the Coulomb part (Def2/J) and a numerical integration grid for the exchange part (DefGrid-2) [50]. Analytical harmonic vibrational frequency calculations were conducted to verify if the ground state was at the minimum on the potential energy surface. To simulate the absorption spectra, the time-dependent density functional theory under the Tamm-Dancoff approximation (TD-DFT/TDA) was employed to obtain the first 30 excitations, using the same calculation protocol [51]. The linear response conductor-like polarizable continuum model (LR-CPCM) method [52] included solvent effects in the excited state energies, adopting acetonitrile as the solvent. The same protocol was used to find transition states with the nudged elastic band method (NEB-TS). All of these calculations were performed using Orca 5.0.4 [53], and the geometric representations of the complexes were obtained using the Chemcraft 1.8 [54].

### 3.3. Preparation of the Copper Complexes

The complexes [Cu(*en*)_2_](ClO_4_)_2_ (**1**), [Cu(*amp*)_2_](ClO_4_)_2_, (**2**) and [Cu(*bpy*)_2_](ClO_4_)_2_ (**3**) (where *en* = ethylenediamine, amp = 2-aminomethylpyridine and *bpy* = 2,2′-bipyridine) were prepared according to previously described procedures [33,34,35]. Their purity was established through elemental analysis, molar conductivity, infrared and electronic spectroscopies and electrochemistry. The FTIR, electronic spectra and cyclic voltammograms are depicted in Figures S1–S3.

#### 3.3.1. [Cu(en)_2_](ClO_4_)_2_ (**1**)

Yield: 48%. Mp 256 °C; Anal. calcd. for C_12_H_16_Cl_2_CuN_4_O_8_: C 12.56, H 4.21, N 14.64, found: C 12.85, H 4.28, N 14.43; UV-Vis (acetonitrile) λ/nm (ε/L mol^−1^ cm^−1^) 231 (8346), 556 (67); IR (ATR) ν/cm^−1^ 3335–3280 (νNH), 2988–2901 (νCH_aliph_), 1590 (δNH), 1113–1009 (νC-C, νC-N); 1095–706 (νCl-O). Molar conductivity (acetonitrile) Λ_M_/ohm^−1^ cm^2^ mol^−1^ 277; Electrochemistry (acetonitrile) *E*_1/2_/V vs. NHE −0.61, *E*_pc_ −1.04.

#### 3.3.2. [Cu(amp)_2_](ClO_4_)_2_ (**2**)

Yield: 87%. Mp 281 °C; Anal. calcd. for C_4_H_16_Cl_2_CuN_4_O_8_: C 30.11, H 3.37, N 11.70, found: C 30.00, H 3.42, N 14.40; UV-Vis (acetonitrile) λ/nm (ε/L mol^−1^ cm^−1^) 252 (12,578), 600 (90); IR (ATR) ν/cm^−1^ 3327–3270 (νNH), 1613–1592 (δNH), 1490–1447 (νC=C, νC=N), 1070–1030 (νC-C, C-N), 1080 (νCl-O), 776 (δCH_ar_), 621 (δC=N); molar conductivity (acetonitrile) Λ_M_/ohm^−1^ cm^2^ mol^−1^ 293; electrochemistry (acetonitrile) *E*_pc_/V vs. NHE −0.63.

#### 3.3.3. [Cu(bpy)_2_](ClO_4_)_2_ (**3**)

Yield: 65%. Mp 310 °C; Anal. calcd. for C_20_H_16_Cl_2_CuN_4_O_8_: C 41.79, H 2.81, N 9.75, found: C 41.27, H 2.84, N 9.52; UV-Vis (acetonitrile) λ/nm (ε/L mol^−1^ cm^−1^) 241 (15,404), 298 (15,177), 306sh, 723 (132), 900 (125); IR (ATR) ν/cm^−1^ 3118–3072 (νCH_ar_), 1602–1445 (νC=C, νC=N), 1077 (νCl-O), 767 (δCH_ar_), 620 (δC=N); Molar conductivity (acetonitrile) Λ_M_/ohm^−1^ cm^2^ mol^−1^ 295; electrochemistry (acetonitrile) *e*_1/2_/V vs. NHE −0.07, *E*_pc_ −0.21, *E*_pc_ −1.12.

### 3.4. Preparation of Composite Catalyst 3-Fe_3_O_4_@SiO_2_

The silica-coated magnetic nanoparticles (Fe_3_O_4_@SiO_2_) were prepared according to the method already described [55]. A 1.0 × 10^−2^ mol L^−1^ solution of complex **3** in acetonitrile was added to a 100 mL flask, and an aliquot of this solution was removed and measured in UV-Vis, in which an absorbance of 1.380 was obtained at λ_max_ = 732 nm. Then, 5 g of Fe_3_O_4_@SiO_2_ was added to this solution and the mixture was stirred at room temperature for 24 h to maximize the complex immobilization. With the aid of a neodymium magnet, the silica-coated magnetic nanoparticles with the incorporated metal complex (**3**-Fe_3_O_4_@SiO_2_) were separated from the fluid solution, and the supernatant had its absorbance measured once again at the same wavelength (A = 0.360). Considering the Beer Law, the immobilization ratio was 4.4%. Composite recovery from the mother solution was 4.3 g (86%).

### 3.5. Homogeneous Catalytic Assays

For the calibration curves, stock solutions were prepared containing benzyl alcohol (BnOH, 15 g L^−1^), benzaldehyde (BA, 12 g L^−1^) and benzoic acid (BzA, 11 g L^−1^) using acetonitrile as a solvent. From the stock solutions, 7 diluted solutions were prepared with concentrations ranging from 13 mg L^−1^ to 267 mg L^−1^ for BnOH, from 0 mg L^−1^ to 157 mg L^−1^ for BA and from 0 mg L^−1^ to 181 mg L^−1^ for BzA using acetonitrile as a solvent. Curves were constructed by injecting 1 μL aliquots of the analyte solution into the GC-MS, using toluene as an internal standard (82 mg L^−1^) to correct instrumental variations. All measurements were carried out in duplicate. The concentration range was calculated based on different compound conversion ranges: BnOH 5% to 100%, BA 0% to 60% and BZA 0% to 60%. The calibration parameters obtained for product quantification were extracted from the GC-MS analysis and are summarized in Table S6.

Oxidation reactions were carried out in screw-capped 4.5 mL amber glass vials with a PTFE-coated silicone septum obtained from Sigma-Aldrich, into which the catalyst (complexes **1**, **2** or **3**), the oxidant (H_2_O_2_ 35% in water) and the substrate (BnOH) solutions were added. The solvent (acetonitrile) was added until the final volume mark of 4.5 mL was reached. The reactions were maintained under constant magnetic stirring at 20 °C. The amount of substrate added in each reaction was fixed at 277 μmol (equivalent to a mass of 30 mg for BnOH), and the amounts of catalyst (mol%) and hydrogen peroxide (nH_2_O_2_/nBnOH) were calculated based on this value.

**Scheme 1:**
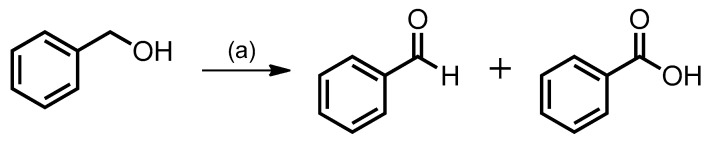
The oxidation reaction of BnOH with H_2_O_2_ is mediated by copper complexes **1**, **2** and **3**. Condition (a): Catalyst 0.1 mol% to 3.0 mol%, H_2_O_2_ 1-fold to 20-fold, time 8 h to 24 h, 20 °C and acetonitrile used as a solvent.

After completing each reaction for the time required by the experimental design, the reaction vials were sonicated for 30 s to remove the oxygen gas formed by the decomposition of the hydrogen peroxide, as its presence inhibited the pipetting of the solution. In the next step, 80 μL of the sonicated reaction mixture was added into a 2.0 mL screw-capped glass vial with a PTFE-coated silicone septum with 320 μL of toluene (internal standard, 82 mg L^−1^) and 1600 μL of acetone (solvent), then injected into the gas chromatograph for the quantification of the products using the areas obtained from the previously prepared calibration curves. Scheme 1 shows the oxidation reaction of the BnOH to afford BA and BzA.

Oxidation reactions with the radical scavenger *N*-oxyl-2,2,6,6-tetramethylpiperidine (TEMPO) were carried out in flasks with a 4.5 mL PTFE septum (Sigma Aldrich, Burlington, VT, USA), in which the catalyst (prepared complexes), oxidant (H_2_O_2_), substrate (benzyl alcohol), TEMPO and dried acetonitrile as a reaction solvent were added up to 4.5 mL (final reaction volume) considering their optimized parameters. The reactions were carried out in triplicate, maintained under magnetic stirring at 25 °C. After completion, each reaction was subjected to an ultrasonic bath to remove the oxygen gas from the peroxide decomposition, diluted in acetone (80 μL of sample and 320 μL of internal standard for a final volume of 2000 μL) and injected into the GC-MS for product quantification.

### 3.6. Separation and Quantification of the Substrate and Products

The separation and quantification of the BnOH and its oxidation products were performed using a gas chromatograph coupled with a quadrupolar-low-resolution mass spectrometer (GCMS-QP2010 Ultra, Shimadzu, Kyoto, Japan) and a 15-slot auto-injector AOC-20i (Shimadzu, Kyoto, Japan). MS-grade helium gas adjusted to 1.20 mL min^−1^, corresponding to a pressure of 63.9 kPa, operated as the mobile phase. A low-bleed HP-5ms column (Agilent Technologies, Santa Clara, CA, USA) was used with the following specifications: 30 m in length, 0.25 mm in internal diameter and 0.25 μm film thickness containing 5%-diphenyl/95%-dimethyl (polysiloxane) as the stationary phase. The injector, interface and ion source temperatures were kept at 250 °C, 250 °C and 200 °C, respectively. The oven temperature was 40 °C for 3 min, followed by a heating rate of 10 °C min^−1^ to 120 °C and then 25 °C min^−1^ to 300 °C, totaling 19.2 min per run. Data acquisition was performed from 3.00 min to 19.20 min in full scan mode, with a range from 40 *m*/*z* to 300 *m*/*z*, considering a 1.0 μL injection and a 1:50 split ratio.

### 3.7. Multivariate Statistical Analysis

The Box–Behnken multivariate design’s experimental outline and chosen variables were based on preliminary studies already published in the literature [30]. A set of fifteen experiments were carried out, considering the catalyst load (metal complexes, mol%), oxidant (H_2_O_2_) and time (h), as shown in Table S7. An initial catalyst concentration of 0.1 mol% was selected, which corresponds to a ratio of 1:1000 concerning the substrate. The highest level was chosen to be 3 mol%, with a center point at 1.55 mol%. The proportion of hydrogen peroxide was 1, 10.5 and 20 times the number of moles of the substrate. The reaction time was evaluated in the 8 h to 24 h range, with the center point at 16 h. The temperature was kept constant at 20 °C.

The regression model generated by the Box–Behnken design had 10 coefficients: the intercept, 3 linear (L) coefficients for each variable, 3 interaction coefficients between two variables (1 L × 2 L, 1 L × 3 L and 2 L × 3 L) and 3 quadratic (Q) coefficients. The generated models were initially validated by analyzing the coefficient of determination *R*^2^, which was expected to be greater than 0.9, and the residuals, which showed the difference between the values predicted by the model and the values obtained experimentally. Residuals were randomly distributed, indicating that the mathematical model used for the regression was adequate. All models to be discussed were evaluated, and outlying assays, with very high residual values, were replicated so that the minimum requisites for the models to be considered valid were satisfied. Residual plots for all models are visualized in the Supporting Information section (Figure S10).

The significance of the regression model’s coefficients was statistically evaluated using Pareto charts. The *x*-axis value in the Pareto charts represented the calculated t-value, which was obtained from the ratio between the coefficient and its standard error estimated by the software. If the calculated *t*-value was more significant than the critical *t*, the coefficient was considered statistically significant for the mathematical model. The critical *t*-value was tabulated as a function of the number of degrees of freedom and the statistical significance chosen for the analysis (95%). The number of degrees of freedom for the regression model was the difference between the number of experiments and the number of coefficients. The greater the number of degrees of freedom, the better the estimation of the coefficients, so a minimum of 3 was required. In the present work, all generated models were validated with at least 5 degrees of freedom.

### 3.8. Heterogenous Catalytic Assays and Catalyst Recycling Studies

The heterogeneous oxidation reactions were carried out in flasks with a 4.5 mL PTFE septum (Sigma Aldrich), in which the **3**-Fe_3_O_4_@SiO_2_ catalyst was suspended in acetonitrile using an ultrasonic bath for 10 min. Then, the oxidant (H_2_O_2_) and substrate (benzyl alcohol) were added until the 3 mL final volume was reached. The reactions were carried out in triplicate, maintained under magnetic stirring at a temperature of 25 °C. After completion, each reaction was subjected to the ultrasonic bath to remove the oxygen gas from the peroxide decomposition, diluted in acetone (80 μL of sample and 320 μL of internal standard stock solution for a final volume of 2000 μL) and injected into the GC-MS for product quantification. The solid was recovered with the aid of a neodymium magnet, washed with acetonitrile and left to dry on the bench. The recovered solid catalyst from the first reaction was used for the study of reuse in oxidation reactions following the same procedure.

## 4. Conclusions

The catalytic properties of three *N*-rich copper complexes were explored regarding the oxidation of benzyl alcohol (BnOH) to benzaldehyde (BA) and benzoic acid (BzA) in the presence of hydrogen peroxide. In the homogenous reactions, maximized conversions of the substrate to their respective products were obtained considering a multivariate analysis approach, and the mathematical models could thoroughly predict the product conversion considering the studied variable intervals. Control tests at the optimal catalytic conditions proved that the ligands’ presence was crucial for tuning the ability of each complex to catalyze reactions. All complexes could also convert BA into BzA in a consecutive reaction. Control reactions adding TEMPO as a radical scavenger suggested that radical species were formed during the reactions catalyzed by complexes **1**, **2** and **3**. Thus, more than one mechanistic pathway is involved in the catalytic oxidation of BnOH promoted by the complexes studied herein. DFT studies suggested that the Cu(II)-OOH species promotes the BnOH to BA oxidation involving only non-radical species before the RTP and both Cu(II)-OOH and [Cu(II)-O•]^+^ for the BA to BzA oxidation. Complex **3** was successfully anchored in Fe_3_O_4_@SiO_2_ magnetic nanoparticles and had its catalytic properties evaluated, allowing the BnOH to be adequately converted into BA in the presence of H_2_O_2_ (heterogeneous catalysis). Multiple uses of the **3**-Fe_3_O_4_@SiO_2_ nanocomposite proved its recyclability, considering that, after three rounds, conversions were 60% higher than a single homogenous run. TEMPO tests did not affect substrate conversions, indicating that no radical species were involved in the catalysis. Future works point towards a deeper analysis of the mechanistic pathways for the catalytic oxidation of the molecules studied herein, supported by experimental evidence and DFT calculations. Studies based on ligand modification to covalently bond the metal complexes into the SiO_2_ inorganic support are in progress to improve catalysts’ immobilization, avoid solvent leaching and thus improve the recyclability of these composites.

## Figures and Tables

**Table 1:** Factors and their levels applied for the catalytic oxidation of benzyl alcohol (BnOH) in acetonitrile, using H_2_O_2_ as an oxidant mediated by complexes **1**, **2** and **3**, respectively. Conversions are given in (%) for the product generated: benzaldehyde (BA) and benzoic acid (BzA) at 20 °C.

Entry	Catalyst/mol%	H_2_O_2_^a^	Time/h	Conversion/%					
				1		2		3	
				BA	BzA	BA	BzA	BA	BzA
1	0.10	10.5	8	0.79	-	5.19	-	1.75	-
2	3.0	10.5	8	2.56	-	4.74	1.26	5.00	-
3	1.55	1.0	8	0.27	-	1.18	-	1.27	-
4	1.55	20	8	2.71	1.33	2.20	-	6.83	-
5	3.0	10.5	24	3.21	-	5.63	1.05	7.37	5.60
6	1.55	1.0	24	0.35	-	0.56	-	1.47	-
7	0.1	10.5	24	0.37	-	2.68	-	5.05	-
8	1.55	20	24	2.52	1.34	6.05	0.62	12.07	5.25
9	0.1	1.0	16	0.48	-	0.37	n.d	0.73	-
10	3.0	1.0	16	0.38	-	0.76	n.d	1.07	-
11	0.1	20	16	1.08	-	1.30	n.d	3.31	-
12	3.0	20	16	3.66	5.93	7.20	1.40	10.85	2.65
13	1.55	10.5	16	4.28	1.05	4.96	1.10	8.02	-
14	1.55	10.5	16	3.87	1.26	4.64	1.05	9.15	-
15	1.55	10.5	16	4.85	1.67	4.81	1.08	9.68	-

^a^ n/substrate. n.d.-not detected.

**Table 2:** Summary of optimum conditions for the catalytic oxidation of BnOH into BA at 20 °C based on the response surfaces for complexes **1**, **2** and **3** and complementary catalytic tests carried out as controls for a thorough reaction rationale. The conversions in brackets (entries 1 to 3) are the theoretical predictions from the mathematical models.

Entry	Complex	Catalyst/mol%	H_2_O_2_/n n_substrate_^−1^	Time/h	Conversion/% (±sd.)		Selectivity/%	TON ^e^ (×10^3^)	TOF ^f^/h^−1^ (×10^3^)
					BA	BzA			
1	**1**	2.20	14.5	16.3	5.94 ± 0.27 [4.76 ± 0.51]	3.72 ± 0.33	62	48.0	3.00
2	**2**	3.00	20.0	24.0	7.75 ± 0.28 [8.68 ± 0.40]	5.24 ± 0.14	60	45.8	1.91
3	**3**	2.50	20.0	24.0	11.20 ± 0.59 [12.60 ± 0.46]	3.28 ± 0.10	78	79.2	3.30
4	**CS** ^a^	2.20	14.5	16.3	4.22 ± 0.04	-	100		
5	**CS** ^a^	3.00	20.0	24.0	7.42 ± 0.16	-	100		
6	**CS** ^a^	2.50	20.0	24.0	7.69 ± 0.77	-	100		
7	-	-	14.5	16.3	0.54 ± 0.36	-	100		
8	-	-	20.0	24.0	0.57 ± 0.33	-	100		
9	**1** ^b^	2.20	14.5	16.3	-	93.6 ± 6.8	100		
10	**2** ^b^	3.00	20.0	24.0	-	71.6 ± 7.4	100		
11	**3** ^b^	2.50	20.0	24.0	-	56.2 ± 1.8	100		
12	- ^b^	-	14.5	16.3	-	-	100		
13	- ^b^	-	20.0	24.0	-	-	-		
14	**1** ^c^	2.20	14.5	16.3	2.26 ± 0.12	-	100		
15	**2** ^c^	3.00	20.0	24.0	3.44 ± 0.29	-	100		
16	**3** ^c^	2.50	20.0	24.0	5.12 ± 0.15	-	100		
17	**1** ^d^	2.20	14.5	16.3	-	42.2 ± 7.9	100		
18	**2** ^d^	3.00	20.0	24.0	-	59.9 ± 6.0	100		
19	**3** ^d^	2.50	20.0	24.0	-	61.9 ± 3.0	100		

^a^ Copper salt, Cu(ClO_4_)_2_·6H_2_O; ^b^ benzaldehyde was used as the substrate; ^c^ 4.0 eq. of TEMPO was added; ^d^ benzaldehyde was used as substrate, and 4.0 eq. of TEMPO was added; ^e^ TON, turnover number, refers to the ratio of mols of a product (BA)/mols of the catalyst; ^f^ TOF, turnover frequency, refers to TON/time (h^−1^); sd. standard deviation (*n* = 3).

**Table 3:** Summary of conditions for the heterogeneous catalytic oxidation of BnOH into BA at 20 °C for **3**-Fe_3_O_4_@SiO_2_ and complementary catalytic tests carried out as a control for a thorough reaction rationale.

Entry	Catalyst/mol%	H_2_O_2_/n n_substrate_^−1^	Time/h	RV ^a^/mL	Conversion/% (±sd.)	Selectivity/%	TON ^d^ (×10^3^)	TOF ^e^/h^−1^ (×10^3^)
					BA			
1	1.00	20.0	24.0	3.0	5.46 ± 0.31	100	97.4	4.06
2	1.00	20.0	24.0	4.5	2.89 ± 0.30	100	-	-
3	1.00	20.0	24.0	9.0	1.21 ± 0.26	100	-	-
4	1.00	20.0	24.0	3.0	7.34 ± 0.33 ^b^	100	130.2	5.42
5	-	20.0	24.0	3.0	-	-	-	-
6	1.00	20.0	24.0	3.0	5.48 ± 0.31 ^c^	100	-	-
7	1.00	20.0	24.0	3.0	3.75 ± 0.50	100	-	-
8	1.00	20.0	24.0	3.0	2.59 ± 0.07	100	-	-

^a^ Reaction volume; ^b^ homogeneous medium; ^c^ 4.0 eqv. of TEMPO was added; ^d^ TON, turnover number, refers to the ratio of mols of a product (BA)/mols of the catalyst; ^e^ TOF, turnover frequency, refers to TON/time (h^−1^); sd. standard deviation (*n* = 3).

## Data Availability

Data are contained within the article and Supplementary Materials.

## Supplementary Materials

The following supporting information can be downloaded at: https://www.mdpi.com/article/10.3390/molecules29112634/s1, Figure S1. FTIR (ATR) spectra of complexes **1**–**3**. Figure S2. UV-Vis spectra of complexes **1**–**3** in acetonitrile. [C] = 1.0 × 10^−4^ mol L^−1^, insets [C] = 1.0 × 10^−2^ mol L^−1^. Figure S3. Cyclic voltammograms of **1**–**3** in acetonitrile. Conditions: Scan rate 100 mV s^−1^ (1) and 300 mV s^−1^ (**2** and **3**). The ferrocene/ferrocinium redox pair was used as standard. Supporting electrolyte: 0.1 mol L^−1^ *n*-Bu_4_NPF_6_; Working electrode: Pt, counter: Pt wire and reference: Ag/Ag^+^. Complexes concentration 5.0 × 10^−3^ mol L^−1^. Figure S4. Size distributions from Fe_3_O_4_ (top), Fe_3_O_4_@SiO_2_ (middle) and **3**-Fe_3_O_4_@SiO_2_ (bottom) obtained by DLS. Figure S5. TEM images from Fe_3_O_4_ (A-B), Fe_3_O_4_@SiO_2_ (C,D) and **3**-Fe_3_O_4_@SiO_2_-(E,F). Figure S6. XRD patterns for Fe_3_O_4_ and Fe_3_O_4_@SiO_2_ nanoparticles. Figure S7. X-ray Energy Dispersive Spectroscopy (EDS) of the composite **3**-Fe_3_O_4_@SiO_2_. Figure S8. Linear correlation between the conversion (%) of benzyl alcohol (BnOH) into benzaldehyde (BA) and reaction volume (mL) reciprocal. Conditions: 1.0 mol% of **3**-Fe_3_O_4_@SiO_2_, 20-fold H_2_O_2_ over BnOH, 24 h, 20 ^o^C. Table S1. Selected bond lengths for the calculated complexes within D3-B3LYP/Def2-TZVP(-f) level of theory, and some crystallographic data values for comparison. Table S2. Attributions of IR bands for complexes **1**–**3** (experimental and calculated within D3-B3LYP/Def2-TZVP(-f) level of theory). Figure S9. Theoretical absorption spectra of **1**–**3** within D3-B3LYP/Def2-TZVP(-f) level of theory in CH_3_CN and convoluted with Gaussians of 25 nm width. Table S3. Data for the TD-DFT excitations within D3-B3LYP/Def2-TZVP(-f) level for complex **1**. For the TD-DFT difference densities between ground and a specified excited state, hydrogens were omitted for clarity, blue indicates decreased and red indicates increased electronic density. Table S4. Data for the TD-DFT excitations within D3-B3LYP/Def2-TZVP(-f) level for complex **2**. For the TD-DFT difference densities between ground and a specified excited state, hydrogens were omitted for clarity, blue indicates decreased and red indicates increased electronic density. Table S5. Data for the TD-DFT excitations within D3-B3LYP/Def2-TZVP(-f) level for complex **3**. For the TD-DFT difference densities between ground and a specified excited state, hydrogens were omitted for clarity, blue indicates decreased and red indicates increased electronic density. Table S6 Calibration parameters obtained for product quantification extracted from the GC-MS analysis. Table S7. Set of experiments considering the Box-Behnken approach for catalytic assays. (Average values calculated with previously determined upper and lower limits). Figure S10. Residual graph obtained by modeling %conversion as a function of catalyst concentration (**1**, top; **2**, middle; **3** bottom), H_2_O_2_ concentration and reaction time with a 10-coefficients quadratic regression equation. R^2^ = 0.97 for **1**, 0.93 for **2** and 0.98 for **3**. Table S8. Cartesian coordinates for the optimized geometry of Cu(II)-OOH specie derived from complex **1**. Table S9. Cartesian coordinates for the optimized geometry of Cu(II)-OOH specie derived from complex **2**. Table S10. Cartesian coordinates for the optimized geometry of Cu(II)-OOH specie derived from complex **3**. Table S11. Cartesian coordinates for the optimized geometry of [Cu(II)-O•]+ specie derived from complex **1**. Table S12. Cartesian coordinates for the optimized geometry of [Cu(II)-O•]+ specie derived from complex **2**. Table S13. Cartesian coordinates for the optimized geometry of [Cu(II)-O•]+ specie derived from complex **3**. Table S14. Cartesian coordinates for the reactants, TS, and products between [Cu(II)-O•]+ species derived from complex **1** and BA. Table S15. Cartesian coordinates for the reactants, TS, and products between [Cu(II)-O•]+ species derived from complex **2** and BA. Table S16. Cartesian coordinates for the reactants, TS, and products between [Cu(II)-O•]+ species derived from complex **3** and BA.
