# Supplementary material for: Homo- and Heterogeneous Benzyl Alcohol Catalytic Oxidation Promoted by Mononuclear Copper(II) Complexes: The Influence of the Ligand upon Product Conversion
Source: Molecules. 2024 Jun 3;29(11):2634. doi: 10.3390/molecules29112634 (PMC11173773; doi:10.3390/molecules29112634)
Supplement: Supplementary file 1 [file molecules-29-02634-s001.zip › molecules-2991827-supplementary.pdf]

Supplementary Information

Homo- and Heterogeneous Benzyl Alcohol Catalytic Oxidation Promoted by Mononuclear Copper(II) Complexes: The Influence of the Ligand upon Product Conversion

Larissa Chimilouski, William H. Slominski, Ana I. Tillmann, Daniella Will, Aaron M. dos Santos, Giliandro Farias, Edmar Martendal, Karine P. Naidek and Fernando R. Xavier

Table of contents (order of appearance)

Figure S1. FTIR (ATR) spectra of complexes **1-3**. **3**

Figure S2. UV-Vis spectra of complexes **1-3** in acetonitrile. [C] = 1.0×10^{-4} mol L^{-1}, insets [C] = 1.0×10^{-2} mol L^{-1}. **4**

Figure S3. Cyclic voltammograms of **1-3** in acetonitrile. Conditions: Scan rate 100 mV s^{-1} (**1**) and 300 mV s^{-1} (**2** and **3**). The ferrocene/ferrocinium redox pair was used as standard. Supporting electrolyte: 0.1 mol L^{-1} *n*-Bu_4NPF_6; Working electrode: Pt, counter: Pt wire and reference: Ag/Ag^+. Complexes concentration 5.0×10^{-3} mol L^{-1}. **5**

Figure S4. Size distributions from Fe_3O_4 (top), $Fe_3O_4@SiO_2$ (middle) and $Fe_3O_4@SiO_2$-**3** (bottom) obtained by DLS. **6**

Figure S5. TEM images from Fe_3O_4 (A-B), $Fe_3O_4@SiO_2$ (C -D) and $Fe_3O_4@SiO_2$-**3** (E-F). **7**

Figure S6. XRD patterns for Fe_3O_4 and $Fe_3O_4@SiO_2$ nanoparticles. **8**

Figure S7. X-Ray Energy Dispersive Spectroscopy (EDS) of the composite $Fe_3O_4@SiO_2$-**3**. **9**

Figure S8. Linear correlation between the conversion (%) of benzyl alcohol (BnOH) into benzaldehyde (BA) and reaction volume (mL) reciprocal. Conditions: 1.0 mol% of **3**-$Fe_3O_4@SiO_2$, 20-fold H_2O_2 over BnOH, 24 h, 20 ºC. **10**

Table S1. Selected bond lengths for the calculated complexes within D3-B3LYP/Def2-TZVP(-f) level of theory, and some crystallographic data values for comparison. **11**

Table S2. Attributions of IR bands for complexes **1-3** (experimental and calculated within D3-B3LYP/Def2-TZVP(-f) level of theory). **11**

Figure S9. Theoretical absorption spectra of **1-3** within D3-B3LYP/Def2-TZVP(-f) level of theory in CH_3CN and convoluted with Gaussians of 25 nm width. **12**

Table S3. Data for the TD-DFT excitations within D3-B3LYP/Def2-TZVP(-f) level for complex **1**. For the TD-DFT difference densities between ground and a specified excited state, hydrogens were omitted for clarity, blue indicates decreased and red indicates increased electronic density. **13**

Table S4. Data for the TD-DFT excitations within D3-B3LYP/Def2-TZVP(-f) level for complex **2**. For the TD-DFT difference densities between ground and a specified excited **14**

state, hydrogens were omitted for clarity, blue indicates decreased and red indicates increased electronic density.

Table S5. Data for the TD-DFT excitations within D3-B3LYP/Def2-TZVP(-f) level for complex **3**. For the TD-DFT difference densities between ground and a specified excited state, hydrogens were omitted for clarity, blue indicates decreased and red indicates increased electronic density. **15**

Table S6. Calibration parameters obtained for product quantification extracted from the GC-MS analysis. **16**

Table S7. Set of experiments considering the Box-Behnken approach for catalytic assays. (Average values calculated with previously determined upper and lower limits) **16**

Figure S10. Residual graph obtained by modeling %conversion as a function of catalyst concentration (**1**, top; **2**, middle; **3** bottom), H_2O_2 concentration and reaction time with a 10-coefficients quadratic regression equation. R^2 = 0.97 for **1**, 0.93 for **2** and 0.98 for **3**. **17**

Table S8. Cartesian coordinates for the optimized geometry of Cu(II)-OOH specie derived from complex **1**. **18**

Table S9. Cartesian coordinates for the optimized geometry of Cu(II)-OOH specie derived from complex **2**. **19**

Table S10. Cartesian coordinates for the optimized geometry of Cu(II)-OOH specie derived from complex **3**. **20**

Table S11. Cartesian coordinates for the optimized geometry of $[Cu(II)\text{-}O\bullet]^+$ specie derived from complex **1**. **21**

Table S12. Cartesian coordinates for the optimized geometry of $[Cu(II)\text{-}O\bullet]^+$ specie derived from complex **2**. **22**

Table S13. Cartesian coordinates for the optimized geometry of $[Cu(II)\text{-}O\bullet]^+$ specie derived from complex **3**. **23**

Table S14. Cartesian coordinates for the reactants, TS, and products between [Cu(II)-O•]+ species derived from complex **1** and BA. **24**

Table S15. Cartesian coordinates for the reactants, TS, and products between [Cu(II)-O•]+ species derived from complex **2** and BA. **27**

Table S16. Cartesian coordinates for the reactants, TS, and products between [Cu(II)-O•]+ species derived from complex **3** and BA. **30**

This is an open-access article distributed under the terms of the Creative Commons Attribution Licence.

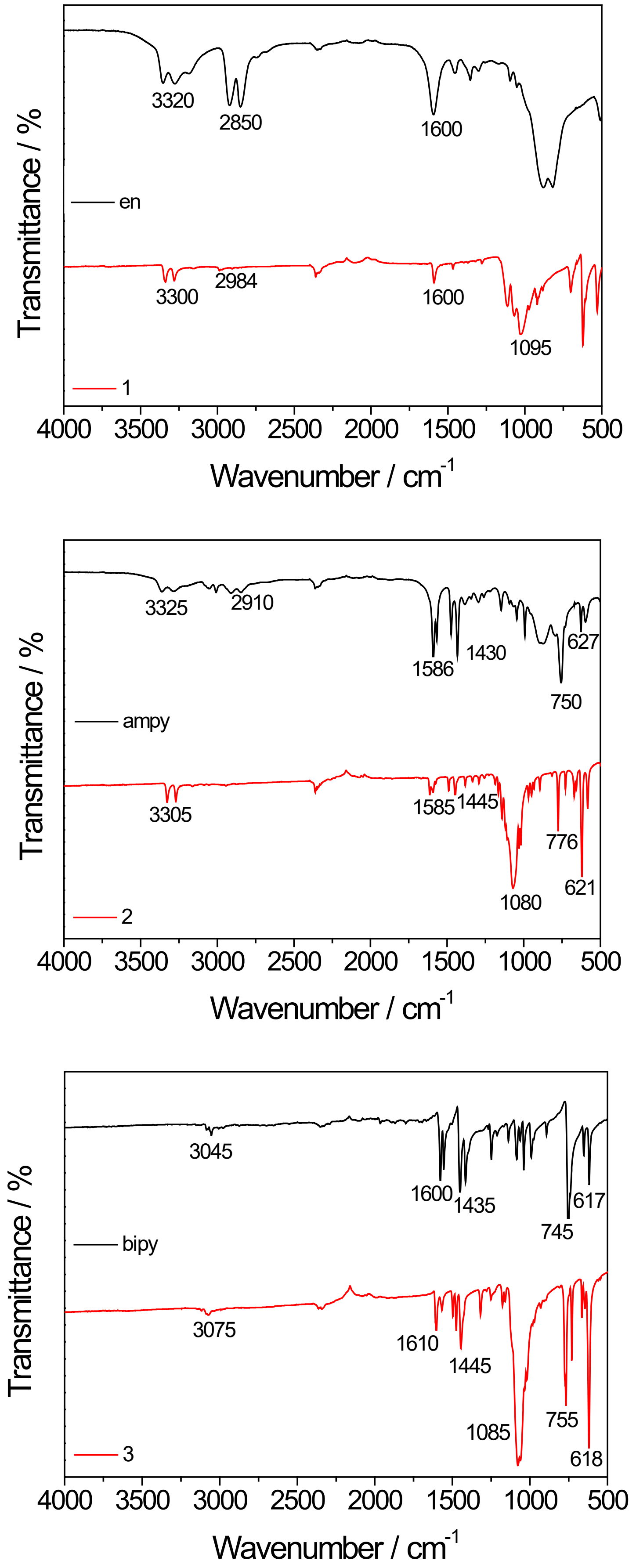

Figure S1. FTIR (ATR) spectra of complexes **1**-**3**.

This is an open-access article distributed under the terms of the Creative Commons Attribution Licence.

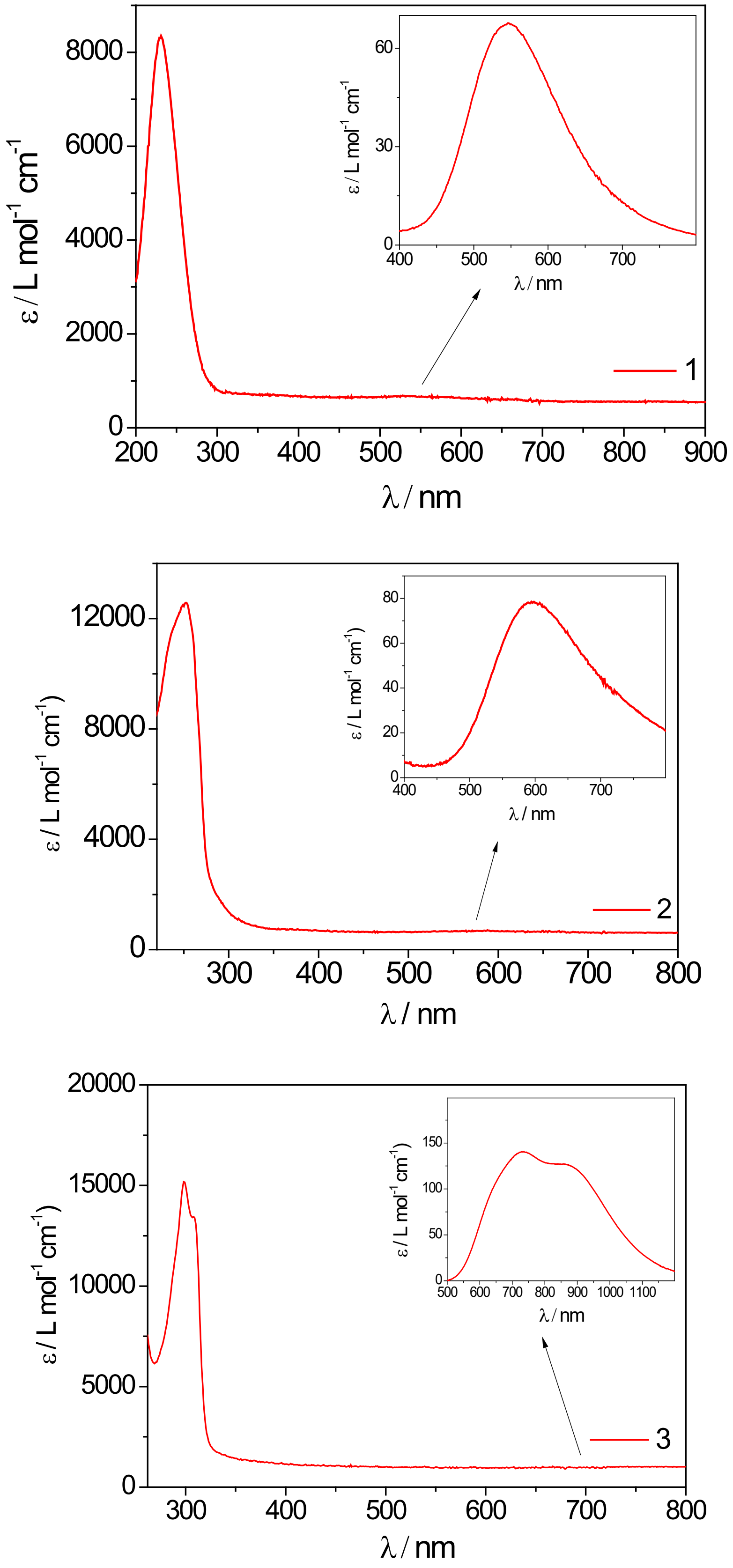

Figure S2. UV-Vis spectra of complexes **1-3** in acetonitrile. [C] = 1.0×10^{-4} mol L^{-1}, insets [C] = 1.0×10^{-2} mol L^{-1}.

This is an open-access article distributed under the terms of the Creative Commons Attribution Licence.

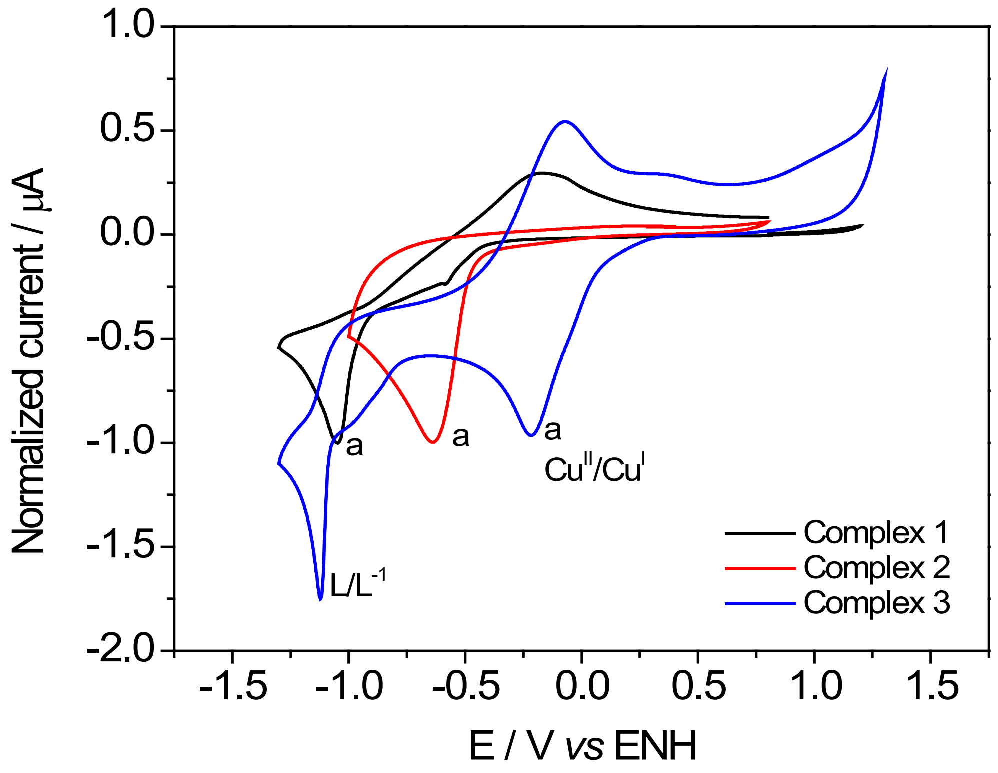

Figure S3. Cyclic voltammograms of **1**-**3** in acetonitrile. Conditions: Scan rate 100 mV s^{-1} (**1**) and 300 mV s^{-1} (**2** and **3**). The ferrocene/ferrocinium redox pair was used as standard. Supporting electrolyte: 0.1 mol L^{-1} *n*-Bu_4NPF_6; Working electrode: Pt, counter: Pt wire and reference: Ag/Ag^+. Complexes concentration 5.0×10^{-3} mol L^{-1}.

This is an open-access article distributed under the terms of the Creative Commons Attribution Licence.

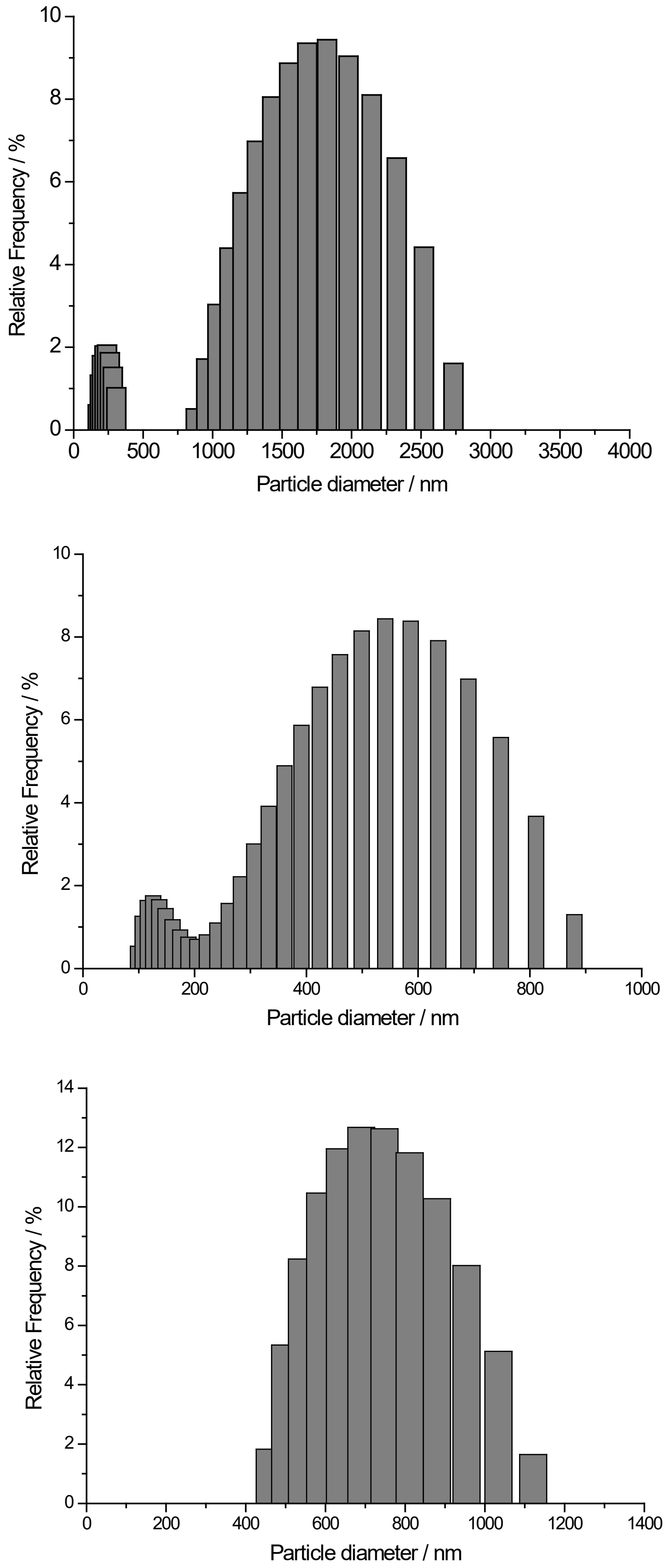

Figure S4. Size distributions from Fe_3O_4 (top), $Fe_3O_4@SiO_2$ (middle) and **3**-$Fe_3O_4@SiO_2$ (bottom) obtained by DLS.

This is an open-access article distributed under the terms of the Creative Commons Attribution Licence.

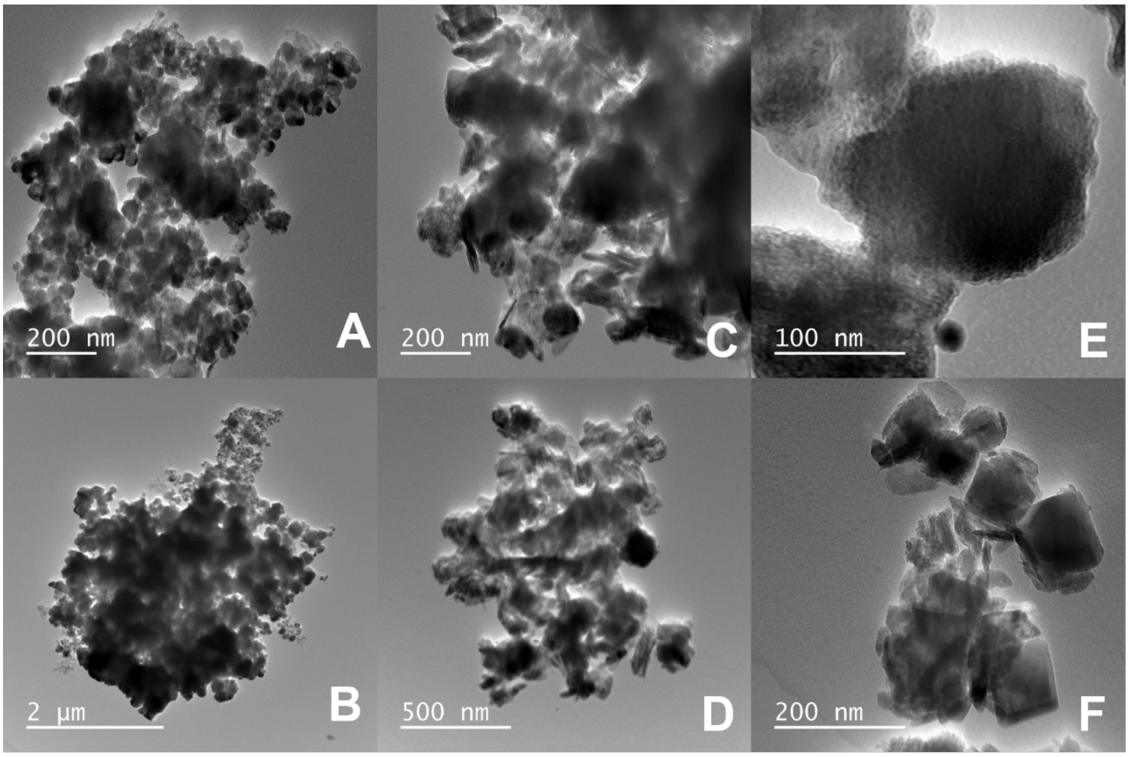

Figure S5. TEM images from Fe_3O_4 (A-B), Fe_3O_4@SiO_2 (C -D) and **3**-Fe_3O_4@SiO_2 (E-F).

This is an open-access article distributed under the terms of the Creative Commons Attribution Licence.

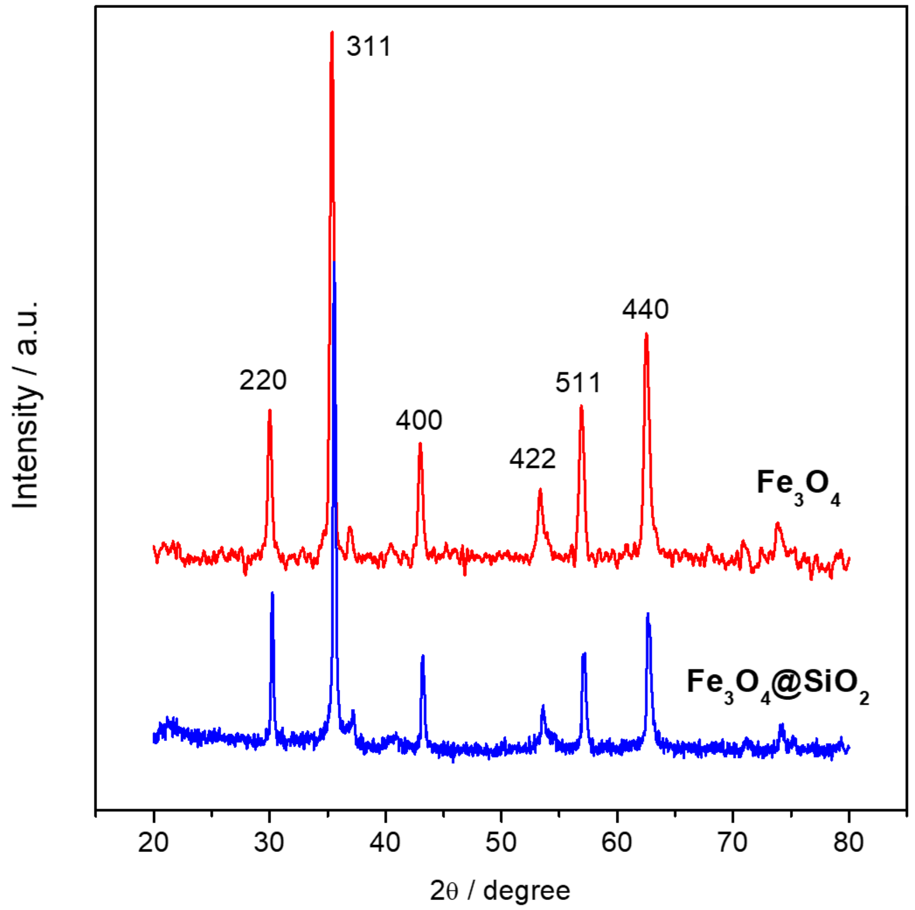

Figure S6. XRD patterns for Fe_3O_4 and $Fe_3O_4@SiO_2$ nanoparticles.

This is an open-access article distributed under the terms of the Creative Commons Attribution Licence.

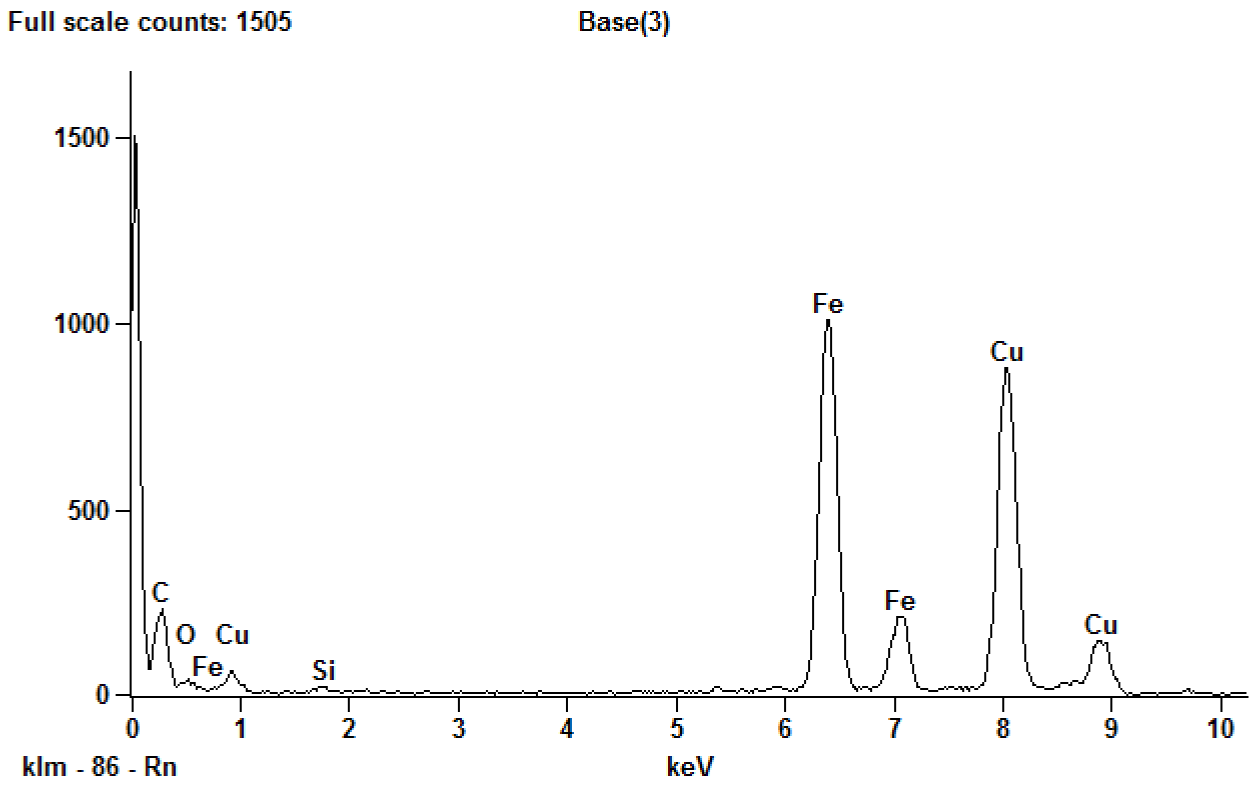

Figure S7. X-Ray Energy Dispersive Spectroscopy (EDS) of the composite **3**-Fe_3O_4@SiO_2.

This is an open-access article distributed under the terms of the Creative Commons Attribution Licence.

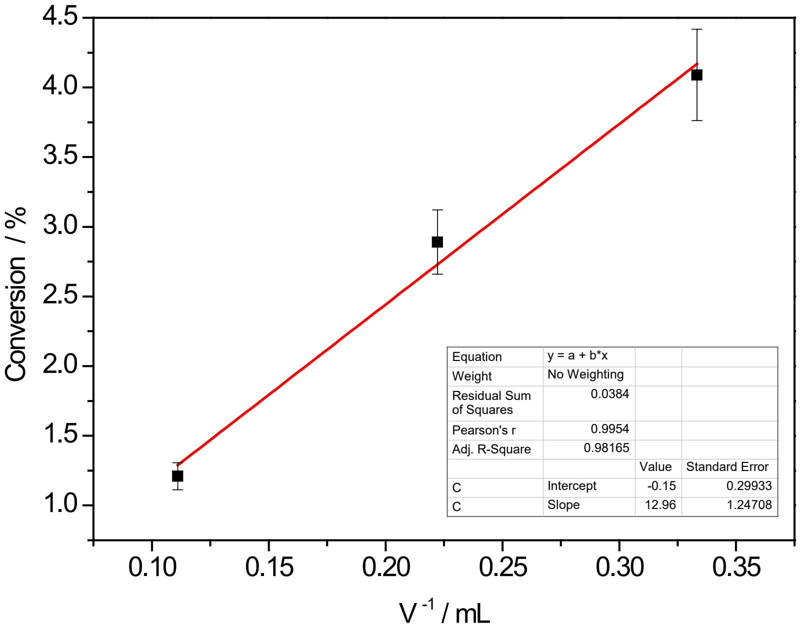

Figure S8. Linear correlation between the conversion (%) of benzyl alcohol (BnOH) into benzaldehyde (BA) and reaction volume (mL) reciprocal. Conditions: 1.0 mol% of **3**-Fe_3O_4@SiO_2, 20-fold H_2O_2 over BnOH, 24 h, 20 ºC.

This is an open-access article distributed under the terms of the Creative Commons Attribution Licence.

Table S1. Selected bond lengths for the calculated complexes within D3-B3LYP/Def2-TZVP(-f) level of theory, and some crystallographic data values for comparison.

Theoretical	1[a]	1 Theo	2 Theo	3[b]	3 Theo
**Cu(II)-N$_1$**	2.002	2.037	2.032	2.004	2.006
**Cu(II)-N$_2$**	2.003	2.038	2.018	1.986	2.002
**Cu(II)-N$_3$**	2.018	2.036	2.030	2.006	2.002
**Cu(II)-N$_4$**	2.003	2.038	2.017	1.979	2.003
**N$_1$-Cu(II)-N$_2$**	84.75	84.55	82.00	81.89	81.81
**N$_1$-Cu(II)-N$_3$**	180.00	176.83	169.87	154.95	152.66
**N$_1$-Cu(II)-N$_4$**	95.25	95.49	98.65	101.74	104.51
**N$_2$-Cu(II)-N$_3$**	95.25	95.48	98.46	102.95	104.69
**N$_2$-Cu(II)-N$_4$**	180.00	177.07	172.80	159.03	153.11
**N$_3$-Cu(II)-N$_4$**	84.75	84.63	82.16	82.57	81.82

Experimental DRX data reported by (a) Altahan, A.M.; Beckett, M.A.; Coles, S.J.; Horton, P.N. Synthesis and characterization of polyborates templated by cationic copper(II) complexes: Structural (XRD), spectroscopic, thermal (TGA/DSC) and magnetic properties. Polyhedron. **2017**, 135, 247-257. (b) Kamp, K.R.; Wang, Y.; Poeppelmeier, K.R. Targeting Oxide Concentration in Tantalum Oxide-Fluoride Anions. Sol. State Sci. **2023**, 146, 107369.

Table S2. Attributions of IR bands for complexes **1-3** (experimental and calculated within D3-B3LYP/Def2-TZVP(-f) level of theory).

Attribution	1<br>Exp. (Calc.)	2<br>Exp. (Calc.)	3<br>Exp. (Calc.)
***v* (N-H)**	3335 - 3280<br>(3539 - 3464)	3327 - 3270<br>(3545 - 3474)	-<br>(-)
***v* (CH$_{ar}$ - CH$_{aliph}$)**	2988 - 2901<br>(3120 - 3063)	-<br>(3225 - 3063)	3118 - 3072<br>(3236 - 3202)
**δ (N-H)**	1590<br>(1629 - 1625)	1613 - 1592<br>(1654 - 1643)	-<br>(-)
***v* (C=C and C=N)**	-<br>(-)	1490 - 1447<br>(1526 - 1484)	1602 - 1445<br>(1648 - 1513)
***v* (C-C and C-N)**	1113 - 1069<br>(1097 - 1009)	1070 - 1030<br>(1035)	-<br>(-)
**δ (C-H$_{ar}$)**	-<br>(-)	776<br>(775 - 747)	767<br>(776 - 752)

This is an open-access article distributed under the terms of the Creative Commons Attribution Licence.

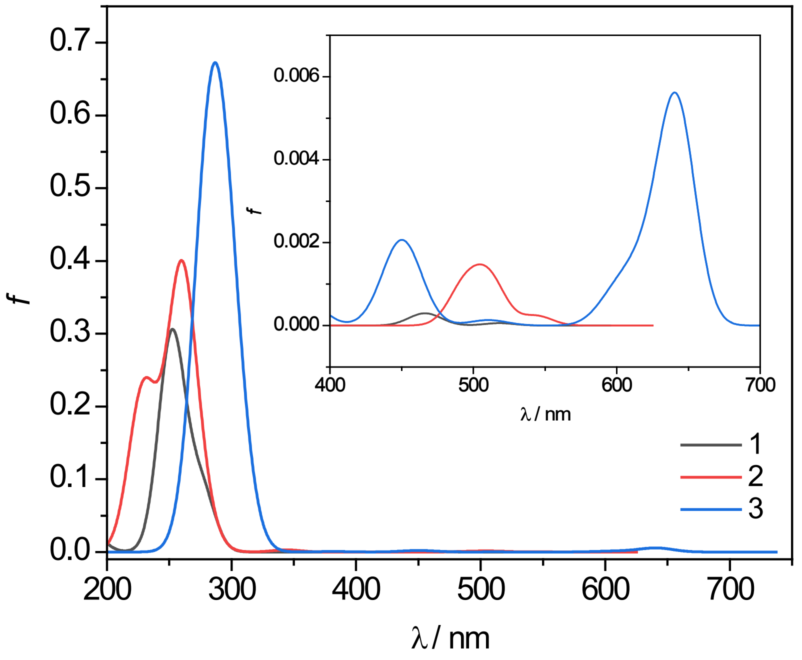

Figure S9. Theoretical absorption spectra of **1-3** within D3-B3LYP/Def2-TZVP(-f) level of theory in CH_3CN and convoluted with Gaussians of 25 nm width.

This is an open-access article distributed under the terms of the Creative Commons Attribution Licence.

Table S3. Data for the TD-DFT excitations within D3-B3LYP/Def2-TZVP(-f) level for complex **1**. For the TD-DFT difference densities between ground and a specified excited state, hydrogens were omitted for clarity, blue indicates decreased and red indicates increased electronic density.

State	Energy		f	Attribution[a]	Difference Densities
	eV	nm			
S_1	2.393	518	6×10^{-5}	$dz^2 \rightarrow dx^2\text{-}y^2$	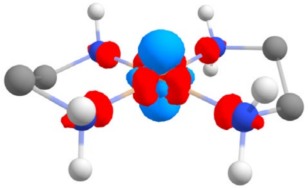
S_2	2.637	470	0.0001	$dxz \rightarrow dx^2\text{-}y^2$	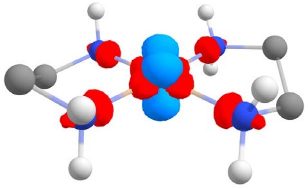
S_3	2.681	462	0.0001	$dyz \rightarrow dx^2\text{-}y^2$	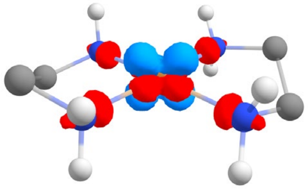
S_4	2.703	458	1×10^{-6}	$dxy \rightarrow dx^2\text{-}y^2$	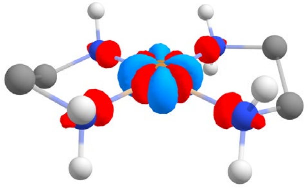
S_5	4.502	275	0.0887	LMCT $n(NH_2) \rightarrow dx^2\text{-}y^2$	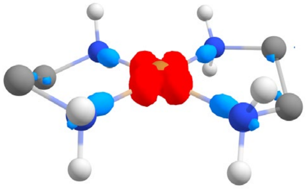
S_6	4.921	252	0.2977	LMCT $n(NH_2) \rightarrow dx^2\text{-}y^2$	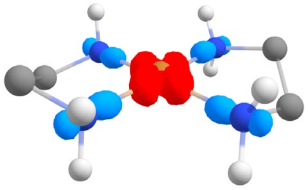

[a] Attributions were given based on the most significant contribution transitions for each excited state.

This is an open-access article distributed under the terms of the Creative Commons Attribution Licence.

Table S4. Data for the TD-DFT excitations within D3-B3LYP/Def2-TZVP(-f) level for complex **2**. For the TD-DFT difference densities between ground and a specified excited state, hydrogens were omitted for clarity, blue indicates decreased and red indicates increased electronic density.

State	Energy		*f*	Attribution[a]	Difference Densities
	eV	nm			
S_1	2.280	544	0.0002	$dz^2 \rightarrow dx^2\text{-}y^2$	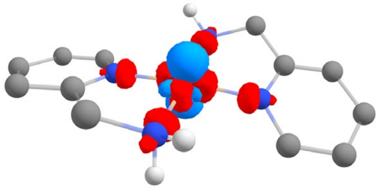
S_2	2.426	511	0.0011	$dxz \rightarrow dx^2\text{-}y^2$	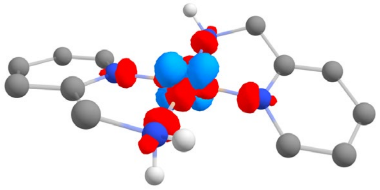
S_3	2.513	493	0.0009	$dyz \rightarrow dx^2\text{-}y^2$	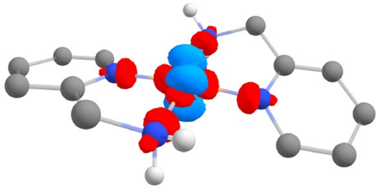
S_4	2.650	468	9×10^{-7}	$dxy \rightarrow dx^2\text{-}y^2$	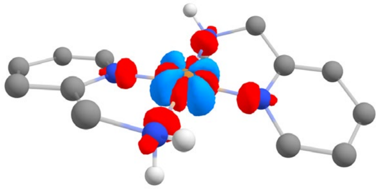
S_{12}	4.464	277	0.0238	LMCT<br>$n(\text{NH}) \rightarrow dx^2\text{-}y^2$<br>MLCT<br>$dx^2\text{-}y^2 \rightarrow \pi^*(\text{pyridine})$	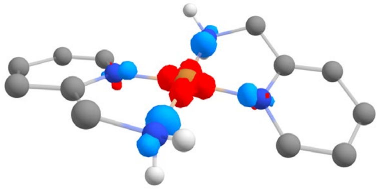
S_{15}	4.701	264	0.1480	LMCT<br>$n(\text{NH}) \rightarrow dx^2\text{-}y^2$	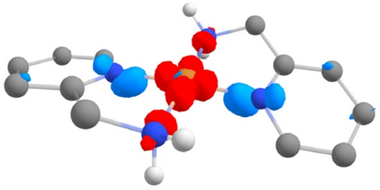
S_{16}	4.821	257	0.2206	LMCT<br>$n(\text{NH}) \rightarrow dx^2\text{-}y^2$	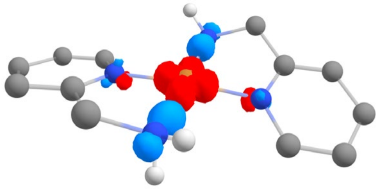

[a] Attributions were given based on the most significant contribution transitions for each excited state.

This is an open-access article distributed under the terms of the Creative Commons Attribution Licence.

Table S5. Data for the TD-DFT excitations within D3-B3LYP/Def2-TZVP(-f) level for complex **3**. For the TD-DFT difference densities between ground and a specified excited state, hydrogens were omitted for clarity, blue indicates decreased and red indicates increased electronic density.

State	Energy		f	Attribution[a]	Difference Densities
	eV	nm			
S_1	1.933	641	0.0053	$dxz \rightarrow dx^2\text{-}y^2$	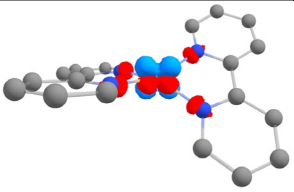
S_2	2.006	618	0.0011	$dz^2 \rightarrow dx^2\text{-}y^2$	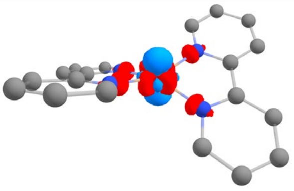
S_3	2.069	599	0.0007	$dyz \rightarrow dx^2\text{-}y^2$	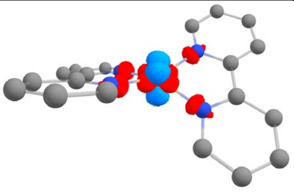
S_4	2.429	510	0.0001	$dxy \rightarrow dx^2\text{-}y^2$	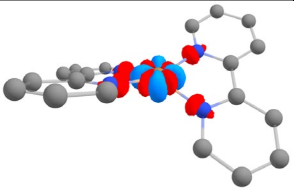
S_{17}	4.176	297	0.1491	LMCT π(pyridine) $\rightarrow dx^2\text{-}y^2$	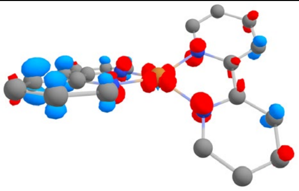
S_{24}	4.339	286	0.1132	π(pyridine) $\rightarrow$ π^*(pyridine)	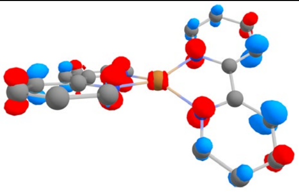
S_{27}	4.437	280	0.1631	LMCT π(pyridine) $\rightarrow dx^2\text{-}y^2$	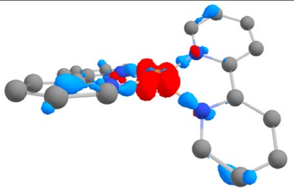

[a] Attributions were given based on the most significant contribution transitions for each excited state.

This is an open-access article distributed under the terms of the Creative Commons Attribution Licence.

Table S6. Calibration parameters obtained for product quantification extracted from the GC-MS analysis.

Compound	Benzaldehyde (BA)	Benzoic acid (BzA)
Linear range (%conversion)	0.20 – 60	0.90-60
LOQ (%conversion)	0.20%	0.90%
R^2	0.9976	0.9861

LOQ = Limit of quantification.

Table S7. Set of experiments considering the Box-Behnken approach for catalytic assays. (Average values calculated with previously determined upper and lower limits)

Entry	Catalyst load / mol%	H_2O_2 / (nH_2O_2/nBnOH)	Time / h
1	0.10	10.5	8
2	3.00	10.5	8
3	1.55	1.0	8
4	1.55	20.0	8
5	3.00	10.5	24
6	1.55	1.0	24
7	0.10	10.5	24
8	1.55	20.0	24
9	0.10	1.0	16
10	3.00	1.0	16
11	0.10	20.0	16
12	3.00	20.0	16
13	1.55	10.5	16
14	1.55	10.5	16
15	1.55	10.5	16

This is an open-access article distributed under the terms of the Creative Commons Attribution Licence.

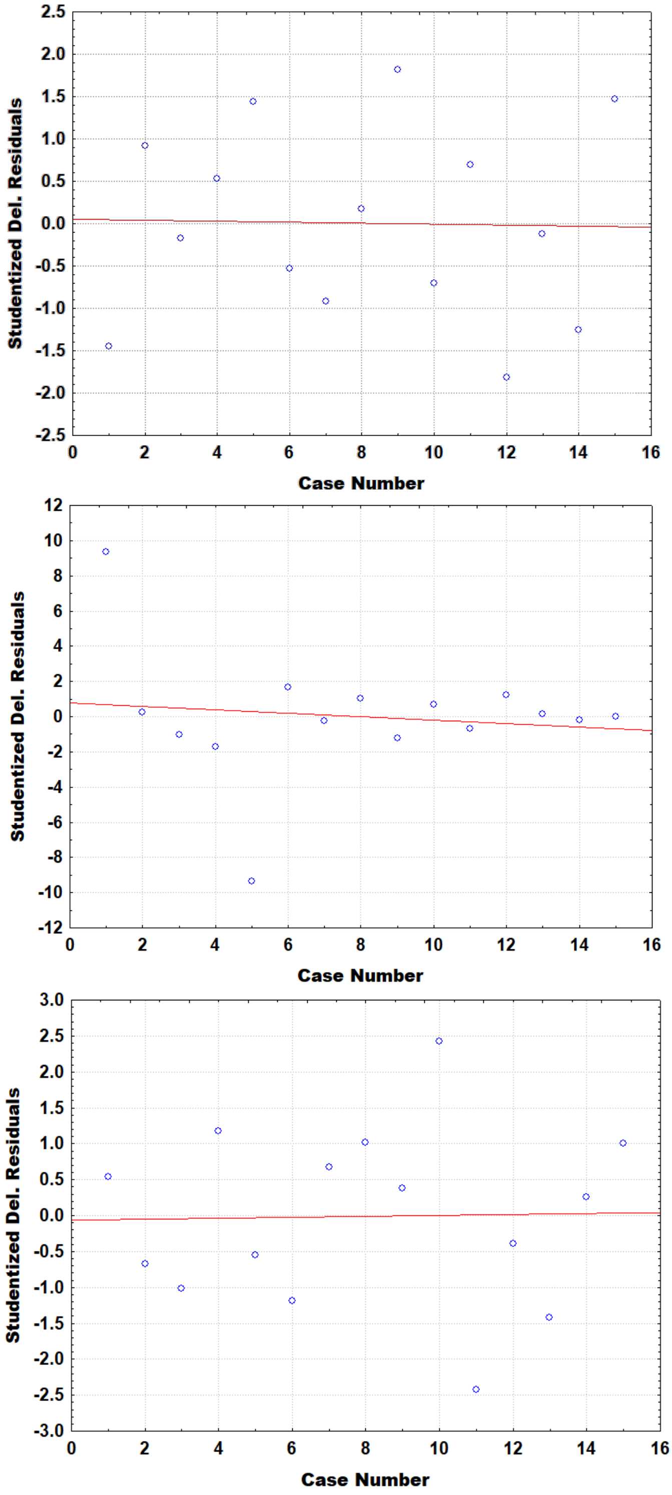

Figure S10. Residual graph obtained by modeling %conversion as a function of catalyst concentration (**1**, top; **2**, middle; **3** bottom), H_2O_2 concentration and reaction time with a 10-coefficients quadratic regression equation. $R^2 = 0.97$ for **1**, 0.93 for **2** and 0.98 for **3**.

This is an open-access article distributed under the terms of the Creative Commons Attribution Licence.

Table S8. Cartesian coordinates for the optimized geometry of Cu(II)-OOH specie derived from complex **1**.

Cu	5.03050842574261	9.15505558443390	6.55157821609912
N	4.74819620951170	10.61115769111753	4.78133143016667
C	5.27871071188184	11.88298928900756	5.28715305953078
C	6.52630042570518	11.62684425118119	6.12662711169254
N	3.83020923040801	7.62491379844082	5.92979597926982
C	2.62789755003655	7.56474665697529	6.77439734990520
C	2.15195765758010	8.99053316579563	7.02877160221127
N	6.21295587127866	10.71208485772580	7.24324090466183
O	6.68276715904840	8.16508014310338	6.32795332222065
N	3.27857806015797	9.77471720287180	7.56969235382469
H	1.84981906521495	9.45241008451905	6.07534647607392
H	1.27583588351571	8.99468518320873	7.69814540850740
H	3.40846412771238	9.57841607636852	8.56512382246103
H	3.09848309124714	10.77707994026587	7.49670345451128
H	5.74361593019892	11.21886313413078	7.99689753416162
H	7.29697633929693	11.14457180343206	5.50477403357498
H	6.93986911986972	12.58263397219149	6.48988128034956
H	2.90512939327980	7.08797298901230	7.72800643496284
H	1.82354442890623	6.96416533265544	6.31851396977921
H	3.58654430862581	7.77957496946297	4.94917509555050
H	5.52297927297898	12.60452602034382	4.48675375667174
H	3.78678013791825	10.71736931058050	4.45746772790493
H	5.29200959810769	10.28823205865619	3.97902634382295
H	4.42279927065846	6.79113375938744	5.96941511388676
H	7.06795071278381	10.32184069584047	7.64275980761201
H	4.50493087790740	12.34944711512014	5.91937528760277
O	6.51729022619019	6.79531337520536	5.89378942882705
H	6.70896691423598	6.85379153896477	4.94245369415610

This is an open-access article distributed under the terms of the Creative Commons Attribution Licence.

Table S9. Cartesian coordinates for the optimized geometry of Cu(II)-OOH specie derived from complex **2**.

Cu	4.16276002992402	10.50743216514085	7.57555043700386
N	3.85982078953139	12.33168922777695	6.67044201296695
C	5.13221587986688	12.81198525129106	6.11173368567553
C	5.93116292754326	11.65108234337114	5.56898000601084
C	6.87120242922570	11.79730149405456	4.54514276608284
H	7.02761078935928	12.77696631495141	4.08904705827486
C	7.58917512276983	10.67848663779939	4.12236338201671
H	8.32999464313959	10.76775852607550	3.32424500231894
C	7.33761476459791	9.44157623338426	4.72368013867998
C	6.37863290682032	9.37662221341712	5.73072854068470
H	6.13830363953872	8.42622082065023	6.21236611857995
H	7.86773851889866	8.53873070806268	4.41577403449760
N	3.01136614957959	8.96536669567386	6.26465012390953
C	2.59457582115450	9.08932618115027	5.00037219521343
C	2.29820201472261	7.98733825330797	4.19668296663911
H	1.95723604086978	8.13089699778345	3.16954030398427
C	2.45886969718845	6.70984641412406	4.74004447368537
H	2.24671701457722	5.82052890874908	4.14142005388052
C	2.90655967897432	6.58344526764362	6.05699792311247
H	3.05666682314626	5.60171256689792	6.51131270053656
H	2.49936811253754	10.10913621132552	4.61343175177083
C	3.16923255159153	7.74307055679671	6.79382530076198
C	3.61263339603066	7.69658902457413	8.24066923557899
N	5.70367784080522	10.45776287423469	6.14591436452523
O	3.15114798924772	11.19712315986608	9.07172723534977
N	4.58982506250013	8.75263399043271	8.55473988516730
H	2.72626999814429	7.87046192045965	8.87278294699263
H	3.99627798238067	6.69542695177809	8.48849169376267
H	5.54780765898966	8.42489990593105	8.42644101779865
H	3.15849605179991	12.22164767175659	5.93434896517769
H	5.70698592879376	13.28239220458185	6.92686776255849
H	4.99070313645232	13.57522738896362	5.33070312960466
H	4.47068572051963	9.04610675606757	9.52939332485876
H	3.47757212502472	12.99512879501096	7.34563909659917
O	3.11364840295412	10.36129829170409	10.24535741758219
H	2.22479236079938	9.96919107521020	10.19783294815628

This is an open-access article distributed under the terms of the Creative Commons Attribution Licence.

Table S10. Cartesian coordinates for the optimized geometry of Cu(II)-OOH specie derived from complex **3**.

Cu	6.19368374834488	5.54398555052999	6.21638119198634
N	7.81646953168238	4.89533720529459	7.28197126874464
C	7.92904018385209	3.66516009636598	7.79348717460878
C	9.03191172227003	3.28419703899059	8.55850051821775
H	9.09309264416283	2.27162931128341	8.96069191488687
C	10.03737839033663	4.22433552124488	8.79071846924864
H	10.91559703441779	3.96494587929638	9.38620398932115
C	9.91163852036257	5.50753480091095	8.25534627144076
H	10.68749703541583	6.25237732098349	8.43006060217838
H	7.10112644322055	2.98813277900163	7.56926317117549
C	8.77756535782272	5.81814044542752	7.49740516362717
C	8.52721395848522	7.14776069980660	6.88956319862095
C	9.39601029822478	8.23684648451746	7.02236258359447
H	10.32263833724148	8.14524429991408	7.58829263230133
C	9.05693854868431	9.45006960386344	6.42201834416311
H	9.72218044070596	10.31129754416942	6.51530847822282
C	7.86115728512750	9.54728641404157	5.70827344671758
C	7.04724975267225	8.41799545912244	5.61930301211883
H	6.10177001133985	8.44328353833448	5.07421584390874
H	7.55671843617120	10.47782330244860	5.22638897929112
N	4.60849229320266	6.44803780405857	7.49033616907835
C	4.61329311359104	6.55907863636213	8.81893587082406
C	3.52313224849638	7.06577166022251	9.52975105858034
H	3.56128502735571	7.14144986130301	10.61797792810750
C	2.39697624447770	7.46668568241804	8.80824540970431
H	1.52102116239298	7.86957231280869	9.32176809836055
C	2.39829041447711	7.35171152492462	7.41710122173330
H	1.52691062703165	7.66904196169481	6.84535865885250
H	5.52168434004580	6.22836898085944	9.33218117100956
C	3.53318763097077	6.83035022352436	6.78000388007507
C	3.63536670277165	6.67902297187545	5.30038477403019
C	2.57836591766732	6.97957848499735	4.43153475777150
H	1.62227361603877	7.33093030607446	4.81748955474863
C	2.75639549461310	6.81658934044809	3.05750664222410
C	3.98598553911153	6.36347043499468	2.57671541629422
C	4.99195134903555	6.08500480468865	3.50182165547936
H	5.97281518338625	5.72415138908640	3.18059209015118
H	4.16824390884948	6.22402585492817	1.50993130692763
H	1.93823409081921	7.04230389126797	2.36989061087054
N	7.37726395764707	7.25606051664557	6.19120017964920
O	5.51220154138029	3.74753856395904	6.13831657366223
N	4.81461675507405	6.23951091225987	4.81717231941420
O	4.08542120487994	3.70294037012287	6.06116025582453
H	3.82178395614242	3.74648021492706	6.99630814225133

This is an open-access article distributed under the terms of the Creative Commons Attribution Licence.

Table S11. Cartesian coordinates for the optimized geometry of [Cu(II)-O•]$^+$ specie derived from complex **1**.

Cu	0.62444401588621	-1.79448609154173	-1.76471079899248
N	-1.08555368920136	-1.28072063080065	-2.84421542983213
N	2.22940345842625	-2.38840962523898	-0.57230321687943
N	-0.37245641226695	-0.82189942702790	-0.21531697600230
N	2.06132121795438	0.05064494635619	-2.11390525975398
C	-1.38929749352310	0.05862239323038	-0.82935010020119
C	-2.09785155977063	-0.72225712424032	-1.92069674163474
H	-2.63884572123460	-1.55923354323539	-1.48073738508155
H	-2.81449148264479	-0.08670692995336	-2.44349519110982
O	1.20264915794747	-2.94522113459511	-3.13444327019897
C	3.43630298612368	-1.63395828541663	-0.97218924705238
H	2.02331691811414	-2.22720140285524	0.40908301653725
C	3.12013795450082	-0.15184437972970	-1.11135967206450
H	2.44343411773504	-0.03455646516689	-3.05021110761949
H	3.76847854500727	-2.03267028852921	-1.93178880975297
H	4.24456830161499	-1.77214559592483	-0.25022186535977
H	2.75699381237441	0.23297236147780	-0.15562973721321
H	4.04226924477855	0.38542659520917	-1.35263266573056
H	1.67198899452916	0.98340387143161	-2.03842087612114
H	2.37487993799037	-3.38876158549923	-0.68657020878614
H	-0.82258610345022	-1.51483349158063	0.37756554833107
H	-2.10988547541634	0.42447191238275	-0.09539845960982
H	-0.86934542317774	0.91725340892096	-1.25619637084649
H	0.25284897132951	-0.28931688929279	0.38028293711555
H	-0.80421636822086	-0.58555264397729	-3.52939552984818
H	-1.45777405191969	-2.07110433804059	-3.35911603804063

This is an open-access article distributed under the terms of the Creative Commons Attribution Licence.

Table S12. Cartesian coordinates for the optimized geometry of [Cu(II)-O•]$^+$ specie derived from complex **2**.

Cu	0.66299865985112	-1.70282422273955	-2.05510948803398
N	-0.82920990730398	-1.05465630103781	-3.33042531644373
N	2.13759342825709	-2.44507842657460	-0.76737954721176
N	-0.69745228694976	-0.97674000028474	-0.66315924853430
N	2.03938344241365	0.17346587781729	-1.65552520014427
C	-1.91452713392605	-0.72594671838175	-1.16496410592269
C	-2.12085402690394	-1.03461758824063	-2.62225792023830
H	-2.56897957620254	-2.02521741898718	-2.70950413377735
H	-2.81646665087133	-0.31873963456534	-3.06207982401497
O	1.50743750114934	-2.61164273513537	-3.46616649584013
C	3.40148540836742	-1.71208664089299	-0.98150510021422
H	1.86883222905795	-2.39479311176241	0.21002070187750
C	3.16777335385446	-0.22217264902160	-1.05909995539621
H	3.81651362182634	-2.05230312101788	-1.93207392516308
H	4.12915910444742	-1.93378157256870	-0.19986050703836
C	-2.93977506494079	-0.22702980524291	-0.37140541579825
H	-0.56696695546772	-0.11814690861437	-3.62491457567847
H	-0.88626211306746	-1.62660050357413	-4.16546786312481
C	-0.45843679473060	-0.72462064031494	0.62892928439149
C	-1.42853078771266	-0.23315734906748	1.48188670357184
C	-2.69716048167033	0.01486790909036	0.97096103531873
H	0.54371985770216	-0.91420197196616	0.98517097595858
H	-3.48229029405288	0.39964883268277	1.60851152047796
H	-1.19074615349834	-0.04856833526044	2.51993551576856
H	-3.90915475923138	-0.03207302847771	-0.81011964046377
C	1.78671309909536	1.47801148240742	-1.77417999358572
C	2.65780650378324	2.45387765775598	-1.31233275133821
C	3.83440368351816	2.04694340053455	-0.69431296593883
H	0.85075990886427	1.74410191943013	-2.25246073071418
C	4.09223083230805	0.68942088228938	-0.55999406352751
H	2.41530955477104	3.50123027688198	-1.42911750251503
H	4.53685983714889	2.77697237517679	-0.31325602331111
H	4.99090277701861	0.33686880346285	-0.07118770455101

This is an open-access article distributed under the terms of the Creative Commons Attribution Licence.

Table S13. Cartesian coordinates for the optimized geometry of [Cu(II)-O•]$^{+}$ specie derived from complex **3**.

Cu	1.05232594362391	-1.68011267967389	-1.58649278569744
N	-0.38608329048589	-1.08911565994853	-2.88595352262312
N	2.50755579038978	-2.10839775631191	-0.18196698031404
N	-0.32005073460221	-0.88645975395365	-0.26667947335218
N	2.29739897594666	0.27046273927237	-1.42831268905825
C	-1.38212248968211	-0.31331139202968	-0.86230648280326
C	-1.42824328288724	-0.44255596277700	-2.33266804105241
C	-2.47163526901677	0.02814917349700	-3.12121711019939
O	1.89848544977903	-2.78994804920950	-2.83015342146564
C	3.38133663276751	-1.14729457242028	0.17361252247381
C	3.19472854598855	0.19425475347462	-0.43593366660147
C	4.41088738262032	-1.42674879689455	1.06954541975893
C	-2.36548819216146	0.32494239116763	-0.11548980794633
C	-0.33897353677319	-1.28967003808896	-4.20299836128101
C	-0.21337173291878	-0.86521376172291	1.06120322001897
C	-1.16342468131993	-0.26173939395156	1.86984055532872
C	-2.25318247020252	0.34957067566250	1.26654205116014
H	0.65592643630734	-1.34823815192925	1.48390819947715
H	-3.01308716774566	0.83625450393257	1.86315915662799
H	-1.04338296126607	-0.27363575786019	2.94380909008409
H	-3.21198578178575	0.78831906349226	-0.59890809193893
C	2.06862415467695	1.43840214236839	-2.02227334723118
C	2.73020390519828	2.60404902997902	-1.65921192335910
C	3.65759950028442	2.53696459670388	-0.62826762670103
H	1.32678597454790	1.43727142328183	-2.81235013855597
C	3.89453290829608	1.32041439718589	-0.00436046784049
H	2.51764158537585	3.53389264862029	-2.16828473339395
H	4.18913442630574	3.42268632540494	-0.30612204343655
H	4.60257291703736	1.26157126617401	0.80842055078274
C	-2.42933335044706	-0.18244109852139	-4.49200483761599
C	-1.34762083538027	-0.85247000655020	-5.04647871710476
H	-3.31040771240240	0.54288787302875	-2.67753309590247
H	0.53074831959719	-1.81590530448637	-4.57217436838629
H	-3.23658180131274	0.17279806356177	-5.11845215878780
H	-1.28087347218747	-1.03752008720050	-6.10913746089216
C	2.62269335084750	-3.33814909513935	0.32559811354667
C	3.61558269814877	-3.67898139772351	1.22831238210710
C	4.52661259393997	-2.70125967244210	1.60272010189127
H	1.89135640707436	-4.06106201982774	-0.00645155469332
H	3.66936047003695	-4.68536535117052	1.61859460717201
H	5.32349471859724	-2.92678774176078	2.29896400861760
H	5.12323642288745	-0.66320729581289	1.34214995199004

This is an open-access article distributed under the terms of the Creative Commons Attribution Licence.

Table S14. Cartesian coordinates for the reactants, TS, and products between [Cu(II)-O•]+ species derived from complex **1** and BA.

Cu	0.62444401588621	-1.79448609154173	-1.76471079899248
N	-1.08555368920136	-1.28072063080065	-2.84421542983213
N	2.22940345842625	-2.38840962523898	-0.57230321687943
N	-0.37245641226695	-0.82189942702790	-0.21531697600230
N	2.06132121795438	0.05064494635619	-2.11390525975398
C	-1.38929749352310	0.05862239323038	-0.82935010020119
C	-2.09785155977063	-0.72225712424032	-1.92069674163474
H	-2.63884572123460	-1.55923354323539	-1.48073738508155
H	-2.81449148264479	-0.08670692995336	-2.44349519110982
O	1.20264915794747	-2.94522113459511	-3.13444327019897
C	3.43630298612368	-1.63395828541663	-0.97218924705238
H	2.02331691811414	-2.22720140285524	0.40908301653725
C	3.12013795450082	-0.15184437972970	-1.11135967206450
H	2.44343411773504	-0.03455646516689	-3.05021110761949
H	3.76847854500727	-2.03267028852921	-1.93178880975297
H	4.24456830161499	-1.77214559592483	-0.25022186535977
H	2.75699381237441	0.23297236147780	-0.15562973721321
H	4.04226924477855	0.38542659520917	-1.35263266573056
H	1.67198899452916	0.98340387143161	-2.03842087612114
H	2.37487993799037	-3.38876158549923	-0.68657020878614
H	-0.82258610345022	-1.51483349158063	0.37756554833107
H	-2.10988547541634	0.42447191238275	-0.09539845960982
H	-0.86934542317774	0.91725340892096	-1.25619637084649
H	0.25284897132951	-0.28931688929279	0.38028293711555
H	-0.80421636822086	-0.58555264397729	-3.52939552984818
H	-1.45777405191969	-2.07110433804059	-3.35911603804063
H	-3.61501252875775	-3.67399103011122	0.64654183788227
H	-3.67512167491793	-4.27209642724543	-1.75300049629348
C	-2.71401718646527	-4.01457306594770	0.15230034624174
C	-2.74990763353380	-4.35246731719387	-1.19741186492538
C	-1.52313747458344	-4.11263402812680	0.87156001743167
C	-1.59275835839595	-4.78599022511765	-1.82947892806956
H	-1.50316981117719	-3.85280809374604	1.92220529391034
H	-1.60489982127240	-5.04358633026517	-2.88195129659130
C	-0.36664998643115	-4.54236650246726	0.24012359442214
C	-0.39640182574707	-4.87775784075921	-1.11624846704143
H	0.56476819244336	-4.62144932037851	0.78498745276990
C	0.81960761068143	-5.32512008526560	-1.81673268706161
O	1.92615418438750	-5.38978119753880	-1.30465133669435
H	0.66397246028367	-5.64725415219891	-2.85761001023281

This is an open-access article distributed under the terms of the Creative Commons Attribution Licence.

Cu	5.30783903700000	5.47729484500000	3.80649395800000
N	3.49033319500000	5.94380378500000	2.79788928800000
N	4.24731861200000	6.30355607000000	5.47308057500000
N	6.64481039400000	7.24364940300000	3.62194679100000
N	6.87361461100000	4.79166281400000	5.10184621800000
C	2.52163890200000	6.46423417600000	3.75229211500000
C	3.24494840800000	7.20085016600000	4.90822907300000
C	7.66266428700000	7.04713256500000	4.67072739100000
C	8.08549732500000	5.58236624400000	4.76144228800000
C	5.47480776100000	1.72776474100000	3.92367148000000
C	4.28927272600000	2.30791919800000	4.62331679600000
C	3.05086753800000	2.38512940900000	3.91823169400000
C	1.90394398800000	2.85639538200000	4.54035335700000
C	1.95695783100000	3.17548098900000	5.90463524000000
C	3.13315541800000	3.03520559200000	6.63350097000000
C	4.31741103000000	2.61551317400000	6.00991944200000
H	3.27452713100000	5.08750228200000	2.32177557900000
H	3.82674033100000	6.63349204400000	2.17235811100000
H	1.98745236700000	5.63153871500000	4.19980672300000
H	1.80810983400000	7.13770088400000	3.26840270200000
H	2.53306524800000	7.50394181100000	5.65606718300000
H	3.82803773200000	8.05991338700000	4.54139671300000
H	3.81886759000000	5.59431675800000	6.02271569400000
H	4.95366116600000	6.76352837700000	6.03107947800000
H	7.11198508200000	7.21977501400000	2.72100410600000
H	6.29015748000000	8.21199908400000	3.65826048100000
H	8.56868858500000	7.68521654400000	4.52598215000000
H	7.28601783700000	7.40892231500000	5.64846572900000
H	8.39802621600000	5.27160759000000	3.78172263200000
H	8.93463869000000	5.46777786500000	5.47510583600000
H	7.06993984000000	3.80709227300000	4.91391183100000
H	6.54757881100000	4.91865817200000	6.04993135600000
H	3.09175911400000	2.18619035300000	2.85945647500000
H	0.92251298200000	2.96111724000000	4.00340218800000
H	1.05643519300000	3.47444578400000	6.38958174100000
H	3.15650173600000	3.27766439500000	7.67274135500000
H	5.28026214200000	2.47722453700000	6.55686926000000
H	6.27566168500000	3.28835273400000	2.53868137000000
O	5.82225285200000	4.14301463200000	2.43917318900000
O	6.64244972900000	1.73188470800000	4.33547951300000

This is an open-access article distributed under the terms of the Creative Commons Attribution Licence.

Cu	0.65300606818443	-1.76401114153500	-1.85662587496684
N	-1.04754575809336	-1.19619040656484	-2.91767572464785
N	2.24303653030489	-2.35156499394233	-0.63240841958028
N	-0.38018341904549	-0.88233969354444	-0.27230920713268
N	2.05982199048945	0.14025445343435	-2.08240133411129
C	-1.39541795613080	0.02746128631319	-0.84501992182665
C	-2.08228814878982	-0.68858646025571	-1.99404796342101
H	-2.63418224416705	-1.54680349267935	-1.61242041126594
H	-2.78730182471330	-0.02224837077495	-2.49401756822416
O	1.18561509109304	-2.84677534285356	-3.35337771871155
C	3.43933896928713	-1.53575970349938	-0.94090635317464
H	1.99273465504377	-2.23971314128684	0.34587219341892
C	3.07139772370092	-0.06296038337629	-1.03306137792496
H	2.49282825151975	0.10119173701298	-2.99942837541349
H	3.83262218821019	-1.87896787566851	-1.89911894140698
H	4.21719117117226	-1.67711428993497	-0.18706104841742
H	2.64222022063732	0.26439269727844	-0.08329413164810
H	3.98253801892558	0.51976854235461	-1.19924492160590
H	1.63581020264247	1.05679487081559	-1.99578649318187
H	2.44820108960361	-3.33750099949694	-0.76499166586422
H	-0.83032231207908	-1.61599736107857	0.26936548780500
H	-2.12759461096637	0.34297971833207	-0.09925633586759
H	-0.87488767079759	0.91336367441723	-1.21087454147221
H	0.22763917796848	-0.38132930414922	0.36714851098096
H	-0.75858903953051	-0.47299913364246	-3.56941143647617
H	-1.38792095938333	-1.97517576771822	-3.47004130167404
H	-3.55168213698338	-3.63376289061521	0.71694801762467
H	-3.64096236716345	-4.13739186161696	-1.70267235891580
C	-2.66427935863307	-3.98516318535227	0.20616207930953
C	-2.71684755611770	-4.26952505102892	-1.15573945002776
C	-1.47653196554838	-4.14920842636918	0.91762138058136
C	-1.58155092658880	-4.72358543771887	-1.81051284420925
H	-1.44376430659461	-3.92862561447836	1.97685064827356
H	-1.60341906383283	-4.94893159884169	-2.86856553625874
C	-0.33601256864507	-4.59300073873849	0.26686716388836
C	-0.38902606712612	-4.88025612438578	-1.10297085571807
H	0.59448389649377	-4.71970553612488	0.80443868374116
C	0.80296248806865	-5.37532444773167	-1.80223326091419
O	1.92719338101280	-5.45710302972062	-1.41501567475225
H	2.04373914657154	-3.24845517523376	-3.18140311681147

This is an open-access article distributed under the terms of the Creative Commons Attribution Licence.

Table S15. Cartesian coordinates for the reactants, TS, and products between [Cu(II)-O•]+ species derived from complex **2** and BA.

Cu	0.66299865985112	-1.70282422273955	-2.05510948803398
N	-0.82920990730398	-1.05465630103781	-3.33042531644373
N	2.13759342825709	-2.44507842657460	-0.76737954721176
N	-0.69745228694976	-0.97674000028474	-0.66315924853430
N	2.03938344241365	0.17346587781729	-1.65552520014427
C	-1.91452713392605	-0.72594671838175	-1.16496410592269
C	-2.12085402690394	-1.03461758824063	-2.62225792023830
H	-2.56897957620254	-2.02521741898718	-2.70950413377735
H	-2.81646665087133	-0.31873963456534	-3.06207982401497
O	1.50743750114934	-2.61164273513537	-3.46616649584013
C	3.40148540836742	-1.71208664089299	-0.98150510021422
H	1.86883222905795	-2.39479311176241	0.21002070187750
C	3.16777335385446	-0.22217264902160	-1.05909995539621
H	3.81651362182634	-2.05230312101788	-1.93207392516308
H	4.12915910444742	-1.93378157256870	-0.19986050703836
H	2.26700731057387	-3.43170822592822	-0.97856509110000
C	-2.93977506494079	-0.22702980524291	-0.37140541579825
H	-0.56696695546772	-0.11814690861437	-3.62491457567847
H	-0.88626211306746	-1.62660050357413	-4.16546786312481
H	-3.51989415926437	-3.34568238808922	1.08136473409840
H	-3.87363306838920	-3.93415684617539	-1.29270158330763
C	-2.70357330824194	-3.75352478458449	0.49950951502521
C	-2.90379405407769	-4.08398039823361	-0.83669523410172
C	-1.45570935304470	-3.94121655074183	1.09382483607309
C	-1.85219636609585	-4.59438908219613	-1.58504359168218
H	-1.30814053302232	-3.68027896650488	2.13333556654646
H	-1.99250780658733	-4.84706798627500	-2.62929519753256
C	-0.40592154202136	-4.45161329805085	0.35064265021921
C	-0.59789189890502	-4.77518924575809	-0.99706569199554
H	0.56744060099924	-4.60140295472397	0.79881012799795
C	0.49978166244026	-5.29948009187079	-1.81888291818258
O	1.65109654834366	-5.43529184060958	-1.43738251039452
H	0.21829615038800	-5.57692807405859	-2.84886135050788
C	-0.45843679473060	-0.72462064031494	0.62892928439149
C	-1.42853078771266	-0.23315734906748	1.48188670357184
C	-2.69716048167033	0.01486790909036	0.97096103531873
H	0.54371985770216	-0.91420197196616	0.98517097595858
H	-3.48229029405288	0.39964883268277	1.60851152047796
H	-1.19074615349834	-0.04856833526044	2.51993551576856
H	-3.90915475923138	-0.03207302847771	-0.81011964046377
C	1.78671309909536	1.47801148240742	-1.77417999358572
C	2.65780650378324	2.45387765775598	-1.31233275133821
C	3.83440368351816	2.04694340053455	-0.69431296593883
H	0.85075990886427	1.74410191943013	-2.25246073071418
C	4.09223083230805	0.68942088228938	-0.55999406352751
H	2.41530955477104	3.50123027688198	-1.42911750251503
H	4.53685983714889	2.77697237517679	-0.31325602331111
H	4.99090277701861	0.33686880346285	-0.07118770455101

This is an open-access article distributed under the terms of the Creative Commons Attribution Licence.

Cu	5.84990662500000	5.09817771000000	4.52891078000000
N	4.22229983000000	5.72252977100000	3.21467641900000
N	4.49403841800000	5.78623952200000	5.88203497100000
N	7.16069375900000	6.89096257900000	4.82708414600000
N	7.34585553800000	4.23904041200000	5.78777626200000
C	2.96795671800000	5.74172935200000	3.99301719900000
C	3.26663715700000	6.12205604800000	5.40732166000000
C	2.29643860500000	6.70430360900000	6.22699954400000
C	2.60464554200000	6.93373470900000	7.57217156600000
C	3.90710021300000	6.64910581000000	8.03484644800000
C	4.79468534400000	6.02397933000000	7.18952742200000
C	6.92120302000000	8.25611861300000	4.80777826500000
C	7.81901873100000	9.19120947000000	5.33107417700000
C	9.00622824700000	8.76431262700000	5.91001127800000
C	9.21635552300000	7.38617519000000	6.00346027900000
C	8.29231208400000	6.48568691300000	5.47329801000000
C	8.57645194100000	4.99760126300000	5.53957091400000
C	5.37073966900000	1.32728228300000	4.90641883500000
C	4.46755796500000	2.09038483000000	5.81829555200000
C	3.17702520100000	2.35079298300000	5.36659811200000
C	2.27016284200000	2.97138633200000	6.19128786600000
C	2.62864485200000	3.27135020000000	7.52233336500000
C	3.91401223700000	2.96756169800000	8.01946992500000
C	4.82513876300000	2.36933982000000	7.15474720600000
H	4.18611940100000	5.09867371000000	2.44750356100000
H	4.55976694800000	6.61115353200000	2.90723297600000
H	2.53423852600000	4.73005289200000	3.96226192700000
H	2.34575906600000	6.55345912200000	3.59174892900000
H	1.34111661800000	6.92659136600000	5.83001953700000
H	1.86507919700000	7.45897532200000	8.19312967700000
H	4.18521691300000	6.85505844200000	9.09954979900000
H	5.83669552600000	5.82006345600000	7.47926363900000
H	6.00433739900000	8.56328682200000	4.32886757200000
H	7.61266521800000	10.22833538700000	5.26776538800000
H	9.67010340000000	9.48203777900000	6.37598662100000
H	10.08416730800000	7.08002510900000	6.56013299100000
H	8.92787027800000	4.71633616800000	4.52050886500000
H	9.30338669100000	4.75124677600000	6.34662399000000
H	7.39305460800000	3.27129838000000	5.46356089100000
H	7.09706062700000	4.32038690300000	6.75956056300000
H	2.91266381800000	2.08881006000000	4.36197343300000
H	1.27206342800000	3.18401864100000	5.82795627300000
H	1.89098172600000	3.70194024300000	8.17718094100000
H	4.17611711300000	3.15777766900000	9.09537435700000
H	5.83097990000000	2.09347296900000	7.46677748200000
H	6.19150388400000	3.58410087200000	2.55185286100000
O	6.81154638700000	4.09483628200000	3.12504081000000
O	6.53772471400000	1.19235989600000	5.18428365300000

This is an open-access article distributed under the terms of the Creative Commons Attribution Licence.

Cu	0.77296190929346	-1.81988313901905	-2.26745842839417
N	-0.76626007403428	-1.15693914026038	-3.47606937290818
N	2.24436025957804	-2.59353058000414	-0.99577600511311
N	-0.51049264292981	-1.05112523614670	-0.82004799925131
N	2.17695549919256	0.05150874254067	-1.86472482822307
C	-1.72914631032838	-0.72914855494806	-1.27677196630066
C	-2.02138868266924	-1.08209993523785	-2.70786577517042
H	-2.49361536685072	-2.06556871521399	-2.73862155740569
H	-2.72368761282852	-0.36624540270908	-3.13639797334624
O	1.68981509720069	-2.74680999604403	-3.67162364768605
C	3.50916963881008	-1.85764885803879	-1.20316983751470
H	2.00623455210941	-2.60856442352935	-0.00932801436985
C	3.28100473380326	-0.36572512641273	-1.23664579826837
H	3.90338043735789	-2.17014785121422	-2.17081721511724
H	4.24700268535054	-2.10532483256418	-0.43913098187288
C	4.18334722323956	0.52742068872586	-0.66879989915169
H	2.36965396925547	-3.56098587797715	-1.27638525066863
C	-2.67506022108223	-0.11947046614556	-0.46343247899134
H	-0.49796243624506	-0.23546352513707	-3.81068786588813
H	-0.88418200029636	-1.74790687332274	-4.29171165501936
H	-3.03209411016232	-3.04661466633301	1.63690823318842
H	-3.76182982188541	-3.54855114686545	-0.66936402007640
C	-2.35768962933458	-3.54882241394687	0.95578969023394
C	-2.77004763826806	-3.83048425875809	-0.34195648624374
C	-1.07794458618001	-3.89907515004206	1.38473193797077
C	-1.90356101061851	-4.46748465057312	-1.21873611288592
H	-0.76440305365511	-3.67093397922274	2.39500884100298
H	-2.20661128037354	-4.69627816258504	-2.23216168032981
C	-0.20484632717893	-4.52810392862758	0.51468121134320
C	-0.61884863897373	-4.81086574996127	-0.79474297827476
H	0.79276115623960	-4.80250100636371	0.83179832954140
C	0.28778467432862	-5.47752099710509	-1.73311211379284
O	1.43968649341728	-5.75377746483163	-1.61089749152089
H	1.34249059186331	-2.45730550161348	-4.52255148544734
C	-0.19447276937523	-0.76705587702685	0.44839915315767
C	-1.08354603671596	-0.16539297116178	1.31953807968234
C	-2.35104543131970	0.16165627220816	0.85424210765936
H	-3.64583470076059	0.13186620029943	-0.86902509239556
H	-3.07263546077044	0.63599020877591	1.50629953957622
H	-0.78322897054897	0.04235902973667	2.33678149758630
H	0.80347087454956	-1.02129332662590	0.77355512236150
C	1.92889771766740	1.35923566342457	-1.94705503399239
C	2.77993359338514	2.31826301703157	-1.41642825012695
H	1.01261224233898	1.64327070374764	-2.45251322206706
C	3.93036642099326	1.88939533341824	-0.76550822272567
H	2.54157925299457	3.36925008336422	-1.50637082219347
H	4.61580148419274	2.60513314515810	-0.33012104837478
H	5.06156430622421	0.15751069713802	-0.15616313219524

This is an open-access article distributed under the terms of the Creative Commons Attribution Licence.

Table S16. Cartesian coordinates for the reactants, TS, and products between [Cu(II)-O•]+ species derived from complex **3** and BA.

Cu	1.05232594362391	-1.68011267967389	-1.58649278569744
N	-0.38608329048589	-1.08911565994853	-2.88595352262312
N	2.50755579038978	-2.10839775631191	-0.18196698031404
N	-0.32005073460221	-0.88645975395365	-0.26667947335218
N	2.29739897594666	0.27046273927237	-1.42831268905825
C	-1.38212248968211	-0.31331139202968	-0.86230648280326
C	-1.42824328288724	-0.44255596277700	-2.33266804105241
C	-2.47163526901677	0.02814917349700	-3.12121711019939
O	1.89848544977903	-2.78994804920950	-2.83015342146564
C	3.38133663276751	-1.14729457242028	0.17361252247381
C	3.19472854598855	0.19425475347462	-0.43593366660147
C	4.41088738262032	-1.42674879689455	1.06954541975893
C	-2.36548819216146	0.32494239116763	-0.11548980794633
C	-0.33897353677319	-1.28967003808896	-4.20299836128101
H	-2.96477816525833	-3.06214099980646	1.39214502516425
H	-3.88289130800630	-2.43495722069415	-0.81553290183756
C	-2.51546609364557	-3.45538616905686	0.48950200293379
C	-3.03294295053220	-3.10103532291033	-0.75156732646786
C	-1.41211707926969	-4.30482625921846	0.57763278738948
C	-2.44106416708925	-3.58807044724525	-1.90910773285211
H	-1.00883679059999	-4.56849212880283	1.54675174866948
H	-2.82232419768321	-3.30243840921638	-2.88236033560103
C	-0.83291986056445	-4.80706049050464	-0.57494499454881
C	-1.34388516038291	-4.44643612590014	-1.82609684673861
H	0.01684894979596	-5.47490729155136	-0.52435460416523
C	-0.75614565468140	-4.96558872691532	-3.06918996498639
O	0.11814716606470	-5.81005339317470	-3.12935809104558
H	-1.18004143584508	-4.53330728440316	-3.99410578871617
C	-0.21337173291878	-0.86521376172291	1.06120322001897
C	-1.16342468131993	-0.26173939395156	1.86984055532872
C	-2.25318247020252	0.34957067566250	1.26654205116014
H	0.65592643630734	-1.34823815192925	1.48390819947715
H	-3.01308716774566	0.83625450393257	1.86315915662799
H	-1.04338296126607	-0.27363575786019	2.94380909008409
H	-3.21198578178575	0.78831906349226	-0.59890809193893
C	2.06862415467695	1.43840214236839	-2.02227334723118
C	2.73020390519828	2.60404902997902	-1.65921192335910
C	3.65759950028442	2.53696459670388	-0.62826762670103
H	1.32678597454790	1.43727142328183	-2.81235013855597
C	3.89453290829608	1.32041439718589	-0.00436046784049
H	2.51764158537585	3.53389264862029	-2.16828473339395
H	4.18913442630574	3.42268632540494	-0.30612204343655
H	4.60257291703736	1.26157126617401	0.80842055078274
C	-2.42933335044706	-0.18244109852139	-4.49200483761599
C	-1.34762083538027	-0.85247000655020	-5.04647871710476
H	-3.31040771240240	0.54288787302875	-2.67753309590247
H	0.53074831959719	-1.81590530448637	-4.57217436838629
H	-3.23658180131274	0.17279806356177	-5.11845215878780
H	-1.28087347218747	-1.03752008720050	-6.10913746089216

This is an open-access article distributed under the terms of the Creative Commons Attribution Licence.

C	2.62269335084750	-3.33814909513935	0.32559811354667
C	3.61558269814877	-3.67898139772351	1.22831238210710
C	4.52661259393997	-2.70125967244210	1.60272010189127
H	1.89135640707436	-4.06106201982774	-0.00645155469332
H	3.66936047003695	-4.68536535117052	1.61859460717201
H	5.32349471859724	-2.92678774176078	2.29896400861760
H	5.12323642288745	-0.66320729581289	1.34214995199004

Cu	6.26486846100000	5.22218132800000	5.80199628100000
N	4.79900686700000	5.75392185000000	4.65430112200000
N	4.94750523700000	6.06006068700000	7.16054596800000
N	7.43649183400000	7.16862796300000	5.93562798100000
N	7.68173777800000	4.83219945400000	7.24664843900000
C	4.80656210400000	5.53787818600000	3.23866209000000
C	3.76531789600000	5.98918058000000	2.44095258400000
C	2.67711472300000	6.66968858200000	3.01046845400000
C	2.64498520200000	6.90116755500000	4.34157072600000
C	3.68696299100000	6.47205729700000	5.15366263400000
C	3.76540770900000	6.64818658400000	6.55361916100000
C	2.83222820700000	7.28810031000000	7.35701340900000
C	2.96532766200000	7.38467812700000	8.72006670900000
C	4.12766357400000	6.74459969600000	9.32152854700000
C	5.00577905400000	6.13242688500000	8.53931299200000
C	7.20878403900000	8.37327251200000	5.36311706500000
C	7.88285657400000	9.54001749100000	5.65196748700000
C	8.84674333000000	9.48172873800000	6.70981024600000
C	9.09945141200000	8.30292285400000	7.35133600300000
C	8.39044076200000	7.14019723500000	7.00415608700000
C	8.58317159400000	5.86831909100000	7.57687827900000
C	9.62987771400000	5.56182961200000	8.49494093900000
C	9.74565358900000	4.32190043500000	9.03726976600000
C	8.80530122900000	3.27291694700000	8.66648195300000
C	7.80209554900000	3.58990421400000	7.75014726900000
C	4.16104700500000	2.20600225900000	4.34990450600000
C	3.64355734500000	2.59987752500000	5.66050190400000
C	2.52842522700000	3.40143153500000	5.72344134300000
C	2.07658171100000	3.88722598000000	6.90132655400000
C	2.72251755900000	3.53443796800000	8.10655480400000
C	3.84741942100000	2.67773186600000	8.02577169000000
C	4.31175690300000	2.20178454000000	6.82465873800000
H	5.66275264800000	5.02752880000000	2.91024941300000
H	3.81044848400000	5.78002503200000	1.39405425500000
H	1.81927362000000	6.99664523700000	2.37450056200000
H	1.81421403300000	7.45505937600000	4.80307958100000
H	1.99402649700000	7.72233404100000	6.88605491700000
H	2.20655476400000	7.84552753100000	9.31043952800000
H	4.21667312200000	6.76424582900000	10.39433125700000
H	5.89684210600000	5.63522779100000	8.94073470600000
H	6.48294451000000	8.36034392000000	4.55030658900000
H	7.66600142800000	10.43161384400000	5.12566048300000
H	9.33076445500000	10.38915207000000	7.00716068800000

This is an open-access article distributed under the terms of the Creative Commons Attribution Licence.

H	9.77868939100000	8.23531225400000	8.18398117600000
H	10.32727158300000	6.35320874500000	8.72775452500000
H	10.56314504300000	4.08310830800000	9.72164258400000
H	8.90636231700000	2.33097622800000	9.10411035800000
H	7.08703180800000	2.89454427100000	7.46147871400000
H	2.04978061400000	3.66133544400000	4.75186076000000
H	1.22526242500000	4.54537526300000	6.93826165900000
H	2.35676813700000	3.89963669800000	8.98148830700000
H	4.36568591700000	2.45789319600000	8.94611871200000
H	5.14494753000000	1.61242897700000	6.74602200700000
H	4.93176821700000	2.80542086800000	3.80714410800000
O	6.96682201600000	4.09986295300000	4.62586779400000
O	5.07373656500000	1.16095246700000	4.20278310700000

Cu	1.07462921124028	-1.92232552520140	-1.72667477418230
N	-0.48617978269282	-1.47054781478065	-2.94849406448532
N	2.59299152302195	-2.22771124181900	-0.36287802500496
N	-0.20729317988627	-0.99359956616965	-0.38603798729955
N	2.31212244678291	0.03828510817175	-1.79424181954756
C	-1.32453028184490	-0.49412395330218	-0.94600026263053
C	-1.49142197553325	-0.77901657248815	-2.38436090558959
C	-2.60213824531427	-0.40147475918657	-3.12983113527789
O	1.80790151111313	-3.13893764919124	-2.99920053068952
C	3.46215837386514	-1.22895990389611	-0.11396040996706
C	3.23397433726678	0.05649093776264	-0.82179598414837
C	4.52347899884284	-1.42028634311875	0.76774367933288
C	3.92055885150966	1.22527328104659	-0.49514570431139
C	-2.24803620421950	0.22182045389310	-0.19238504501428
H	-2.32384317038811	-3.15048411641688	1.84953571686069
H	-4.11282220371166	-2.60435722893937	0.23463396667880
C	-2.23876718133010	-3.47547687790338	0.82077547576186
C	-3.24686747493650	-3.16743983325917	-0.08645255164455
C	-1.11018968345652	-4.18547458085176	0.41203697191847
C	-3.13312901991579	-3.57561359136686	-1.40850266263211
H	-0.32448320751557	-4.40688990477203	1.12252151844947
H	-3.90299907942777	-3.33975643856398	-2.13120264092245
C	-0.98737262777645	-4.59328579555102	-0.90489026810863
C	-2.00629739730206	-4.28919729752868	-1.81874334404275
H	-0.11291467557656	-5.13413297243196	-1.24062980600443
C	-1.90103938471663	-4.70898982973151	-3.21971908938688
O	-1.03976710115957	-5.31916705255212	-3.76912134094921
H	2.68506315245326	-3.40384313781455	-2.70163290647031
C	0.01448206823172	-0.81558322377662	0.91607748781360
C	-0.86569253276901	-0.12085708111317	1.72995558945649
C	-2.01399346446545	0.41102649593923	1.16064221635140
H	-3.14039441823882	0.62063953311683	-0.65023098259248
H	-2.72367812080294	0.96294681091193	1.76225997569516
H	-0.64862874313184	-0.00325848420336	2.78212329288971
H	0.92062434780805	-1.24695224290749	1.31604982550222
C	2.04361430464081	1.15366085435913	-2.46762603648718
C	2.68810823817355	2.35637508590133	-2.21012743045233

This is an open-access article distributed under the terms of the Creative Commons Attribution Licence.

H	1.28351315710578	1.07851287312908	-3.23649647465904
C	3.64212237962014	2.38634337402945	-1.20211676644859
H	2.44247112467863	3.24059155408448	-2.78182109094938
H	4.16196635846270	3.30440144610134	-0.96162529814155
H	4.64894279186055	1.24122368060374	0.30135017351298
C	-0.53833511444149	-1.80947207631391	-4.23527966607776
C	-2.66226383701847	-0.75136602258436	-4.47131133908875
C	-1.61670672805434	-1.46768244013084	-5.03711783602603
H	0.30757931187939	-2.37593681789401	-4.59887417926682
H	-3.52154374903471	-0.46956632234143	-5.06520840325310
H	-1.63166656903157	-1.76273663141185	-6.07676080347356
H	-3.41336208185318	0.14744047556055	-2.67606632673258
C	2.74640756853531	-3.41156760129096	0.23662480135172
C	3.77318335063648	-3.66431363355389	1.13038052067281
C	4.67717005311871	-2.64550694790336	1.39738252050087
H	2.01956899914411	-4.17154407808023	-0.01425420140785
H	3.85753382994148	-4.63535623452242	1.59723403186433
H	5.49833598534283	-2.80242440653871	2.08417732794184
H	5.23044496026982	-0.62669573320706	0.95597300081164

This is an open-access article distributed under the terms of the Creative Commons Attribution Licence.
